# Crosstalk between miRNAs and their regulated genes network in stroke

**DOI:** 10.1038/srep20429

**Published:** 2016-02-02

**Authors:** Ye Yuan, Ruixia Kang, YaNan Yu, Jun Liu, YingYing Zhang, ChunFeng Shen, Jie Wang, Ping Wu, ChunTi Shen, Zhong Wang

**Affiliations:** 1 Nanjing University of Chinese Medicine, 138 Xianlin, Nanjing 210023, China; 2 Institute of Basic Research in Clinical Medicine, China Academy of Chinese Medical Sciences, No. 16 Nanxiaojie, Dongzhimennei, Beijing 100700, China; 3 Changzhou Hospital of Traditional Chinese Medicine, Nanjing University of Chinese Medicine, Heping North Road, Tianning District, Changzhou 213003, China; 4 Guang’anmen Hospital, China Academy of Chinese Medical sciences, Beijing 100053, China

## Abstract

In recent years, more and more studies focus on the roles of genes or miRNAs in stroke. However, the molecular mechanism connecting miRNAs and their targetgenes remains unclear. The aim of this study was to determine the differential regulation and correlations between miRNAs and their targetgenes in human stroke. Stroke-related miRNAs were obtained from the Human MicroRNA Disease Database (HMDD) and their targetgenes were generated from three independent sources. Kappa score was used to create the network and the functional modules. A total of 11 stroke-related miRNAs were identified from the HMDD and 441 overlapping targetgenes were extracted from the three databases. By network construction and GO analysis, 13 functional modules, 186 biological processes, and 21 pathways were found in the network, of which functional module 8 was the largest module, cellular-related process and phosphate-related process were the most important biological processes, and MAPK signaling pathway was the most significant pathway. In our study, all miRNAs regulate the stroke modular network by their targetgenes. After the validation of miRNAs, we found that miR-605 and miR-181d were highly expressed in the blood of stroke patients which never reported before may supply novel target for treatment.

Stroke is the third leading cause of death and long-term disability worldwide. The complexity of the disease resulting from its multiple underlying risk factors has impeded both diagnosis and potential therapy. Until now, the diagnostic and prognostic powers are very limited in stroke management. Therefore, it is imperative to clarify the mechanism of stroke to provide implications for new preventive and therapeutic options.

MicroRNA (miRNA) is small non-coding RNA which regulates at least 60% of all protein-coding gene expression, the most affected genes by miRNAs should be measured in patients with Stroke^1^,^2^. MiRNAs exhibit functional dysregulation in almost all aspects of human pathology, including stroke. Recent studies reported distinct miRNA expression patterns in stroke pathogenic process, including hyperlipidemia, hypertension and plaque rupture and artherosclerosis^3^,^4^. Besides, circulating miRNA expression varies significantly in stroke patients as well as for the different stroke subtypes^5^.

To understand the complex regulations in stroke, researchers have focused on the miRNA genes network. Several studies have reported on the altered expression of miRNAs and their targetgenes during stroke. One study found that several miRNAs were differentially expressed in blood cells of patients with acute ischemic stroke, and these miRNAs were predicted to regulate several genes in pathways previously identified by gene expression analyses^6^. Another study constructed a miRNA-miRNA network using predicted targetgenes of mouse miRNAs microarray expression profiles data to investigate the complex synergistic relationships between miRNAs, identify potential miRNA or targetgenes, and search out the associated biological processes^7^.

These studies have partially revealed the functions of miRNAs and targetgenes in stroke. To date, there is a lack of studies that can fully reveal the roles of biological processes, genes, and miRNAs in human stroke miRNAs network. In this study, we aim to identify stroke-related miRNAs and their targetgenes, and then construct a miRNA-targetgenes functional network to find out important biological processes and determine which miRNAs have major roles in stroke. These analyses may provide a theoretical basis for further studies and help to uncover the complex mechanisms underlying stroke.

## Results

### Identification of stroke-related miRNAs and targetgenes

A total of 11 miRNAs (hsa-mir-122, hsa-mir-124, hsa-mir-133a, hsa-mir-145, hsa-mir-155, hsa-mir-181a, hsa-mir-298, hsa-mir-362, hsa-mir-497, hsa-mir-1, and hsa-let-7f) were identified to be related to human stroke based on the HMDD. Of these, 7 miRNAs (hsa-mir-122, hsa-mir-124, hsa-mir-133a, hsa-mir-145, hsa-mir-155, hsa-mir-181a, and hsa-mir-362) could predict targetgenes in all the three databases. Totally, 445 overlapping targetgenes of the 7 miRNAs among the three databases were found. miR-124 had the largest proportion of targetgenes, accounting for 67.57%, followed by miR-181a and miR-155, accounting for 10.11% and 8.76%, respectively (Supplementary Table 1).

### miRNAs targetgenes network construction

**Figure 1:**
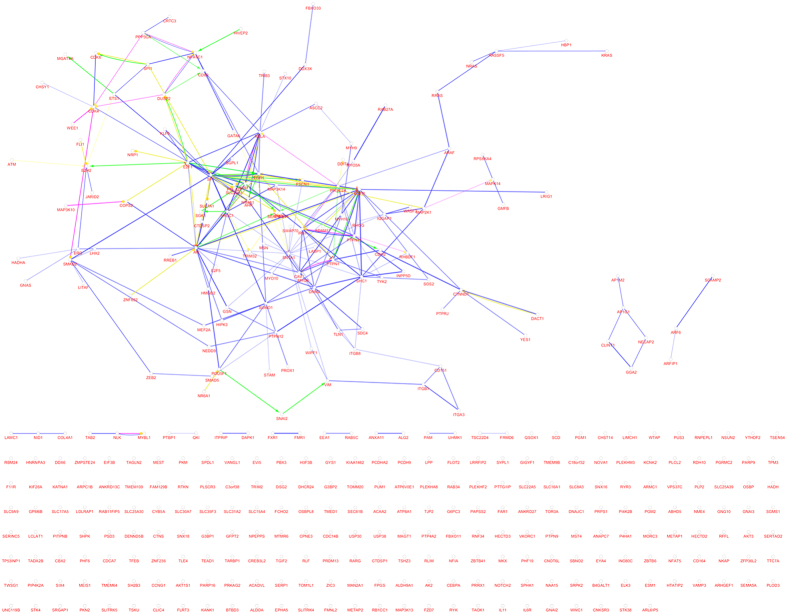
The gene network of stroke. It consists of 379 nodes and 491 edges, indicating that there are 491 connections among the 379 genes.

To investigate the correlations among the targetgenes of each miRNA in a whole network, we combined the targetgenes of the 7 miRNAs and obtained an allied network. Because 66 genes did not have any GO terms (p < 0.05) and were omitted from the network, 379 genes and 186 GO terms were involved in the network (Fig. 1). The whole network had 565 nodes (including 379 genes and 186 GO terms), which were connected by 12,324 edges. The clustering coefficient was 0.366, the number of multi-edge node pairs was 22, and the number of isolated node was 0.The gene IDs, aliases and functions of initial targetgenes are listed in Supplementary Table 2. SP1, AR, EGFR had the largest number of edges in the network, which were 27, 23 and 21, respectively.

### GO analysis of modules based on miRNAs targetgenes network

**Figure 2:**
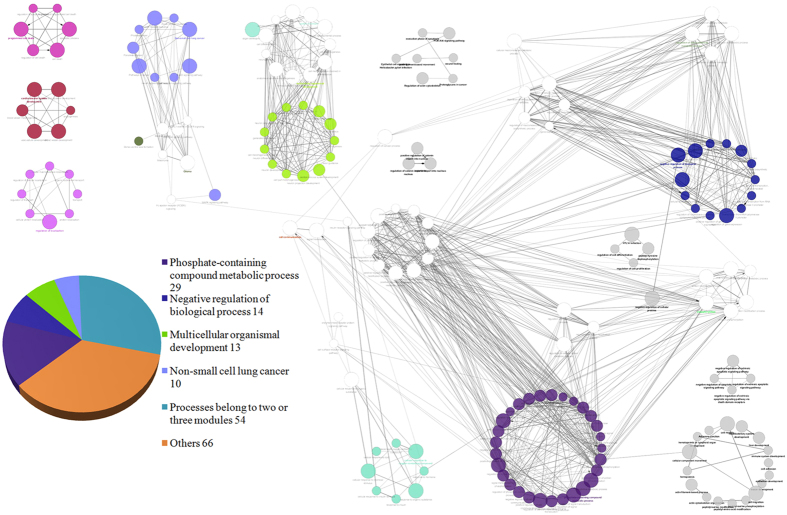
Functional modules in the network. The layout of similar biological processes is presented as a circle. Each color represents one function. For example, the blue colored module is named according to its most significant process, negative regulation of biological process. As one process may involve in several modules, it may not be shown in all of these modules, such as the process of multicellular organismal development.

We analyzed and enriched the genes with publicly available information from STRING. A total of 186 GO terms were found through the annotation, and the number of GO term connections was 491. According to the method discussed above, 13 functional modules, consisting of the most correlated genes and biological functions, were identified (Fig. 2).

Functional module 7, module 11, and module 8 had the largest number of GO terms, which were 55, 38, and 24, respectively. Their most significant functions were phosphate-containing compound metabolic process, negative regulation of cellular process, and multicellular organismal development, respectively (Supplementary Table 3).

Functional module 11, module 7, and module 2 had the largest number of genes, which were 326, 318, and 275, respectively. The most significant function of module 2 was cell communication. Besides, functional module 1 and functional module 12 had 249 and 233 genes, and their most significant functions were regulation of macromolecule metabolic process and phosphorylation, respectively (Supplementary Table 4).

There were 28 cellular-related processes and 12 phosphate-related processes in the network, which were considered the most common biological processes. The most significant functions of cellular-related process were cellular response to lipid, regulation of cellular macromolecule biosynthetic process, and cellular protein modification process.

**Figure 3:**
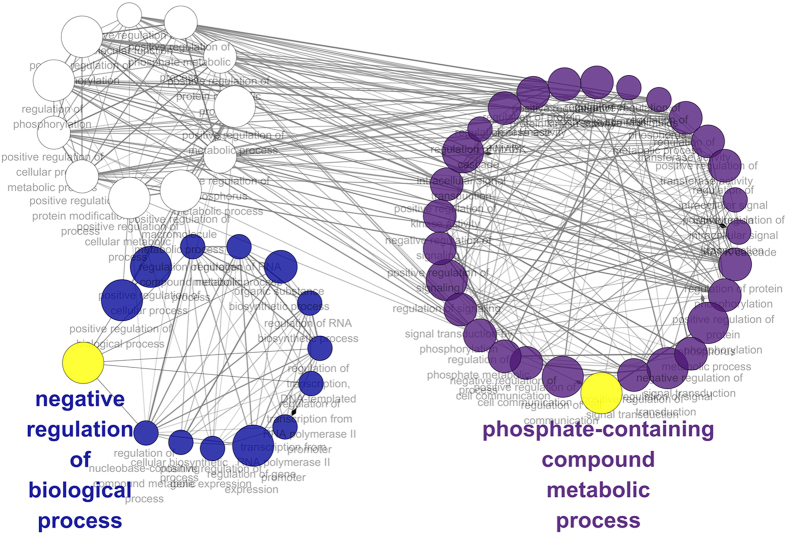
The two largest functional modules and their biological processes. The purple colored biological processes belong to module 7 only, the blue colored biological processes belong to module 11 only, and the white colored biological processes belong to both module 7 and module 11.

In our study, the largest functional module–module 8 had 29 unique and 26 overlapping biological processes compared with functional module 1, 2, 9, 11 and 13. Among the 26 common biological processes, 11 were shared with functional module 11, the second largest functional module in the network (Fig. 3).

### KEGG pathway and miRNAs

There were 21 pathways in the network. Functional module 6 and 5 had the largest number of pathways, which were 10 and 5, respectively. The top 3 most significant pathways were MAPK signaling pathway, regulation of actin cytoskeleton, and FoxO signaling pathway (Table 1). The targetgenes of miR-124 accounted for 40.0%, 31.6%, and 29.4% of all the genes associated with the three pathways, respectively (Table 2). miR-362, miR-133a, and miR-122 had no targetgenes in the three pathways.

### Targetgenes distribution in functional modules

All the miRNAs seemed to be involved in the 13 functional modules by their targetgenes. As miR-124 had the largest number of targetgenes, it might play important roles in the whole network. The targetgenes of miR-124 were involved in all the 13 functional modules.

The three largest functional modules were functional module 11, 7, and 2, which had 209, 229, and 175 targetgenes, respectively. The proportions of targetgenes of miR-124 in the top three functional modules were 64.11%, 72.01%, and 63.63%, respectively.

The most significant terms of targetgenes were multicellular organismal development (in functional module 10 and 8), negative regulation of biological process (in functional module 11), and phosphate-containing compound metabolic process (in functional module 7).

miR-181a had the second largest number of targetgenes. The proportions of targetgenes of miR-181a in the top three functional modules were 11.7%, 11.0%, and 12.0%, respectively. miR-155 had the third largest number of targetgenes. The proportions of targetgenes of miR-155 in the top three functional modules were 10.7, 11.0%, and 12.0%. respectively.

miR-362 had the least number of targetgenes, which were PTPN1 and E2F1. However, they were also involved in all the 13 functional modules. The functions of PTPN1 were peptidyl-tyrosine dephosphorylation and adherens junction. The functions of E2F1 were enzyme linked receptor protein signaling pathway and positive regulation of gene expression.

### Crosstalk among miRNAs targetgenes and functional pairs

**Figure 4:**
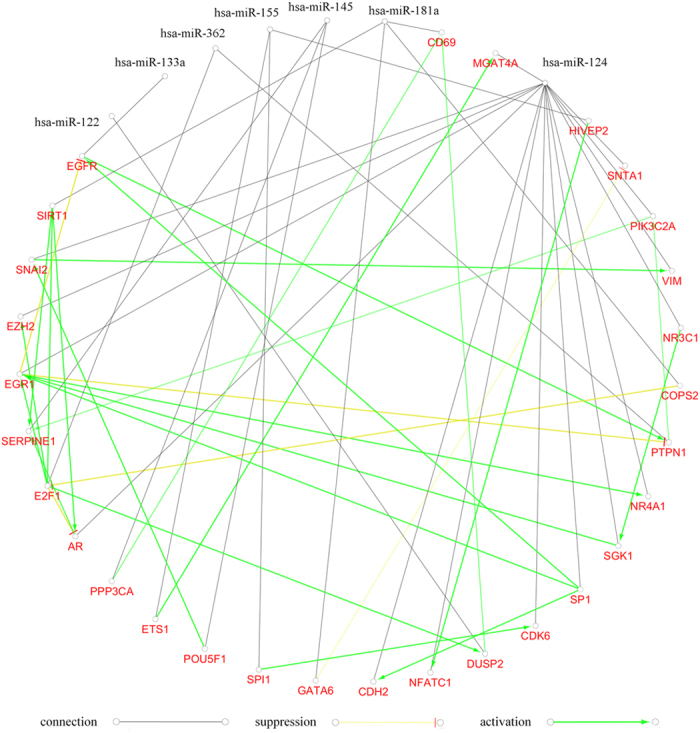
Targetgenes of miRNAs and their correlations. (green color denotes active, yellow denotes suppressive, and black denotes connective)

miRNAs were allied by targetgenes. As an upstream regulator, a miRNA could influence another miRNA and biological process by its effector, targetgenes. For instance, SIRT1, which was regulated by miR-181a, was also activated by E2F1. E2F1 was regulated by miR-362, and it was also suppressed by COPS2, a targetgene of miR-181a. Therefore, the correlations among SIRT1, E2F1, and COPS2 constructed a bridge between miR-362 and miR-181a. The whole network had 15 active gene pairs (one gene can enhance the other gene expression) and 5 suppressive gene pairs (one gene can decrease the other gene expression). Twenty-nine genes got involved in the activation and inhibition directly, 14 of which were targetgenes of miR-124 (Fig. 4).

### Related miRNA prediction

**Figure 5:**
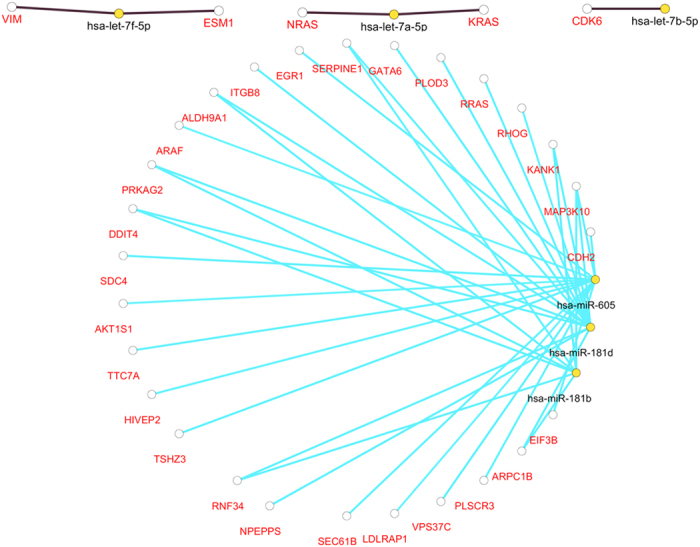
Predictive miRNAs and their targetgenes. let-7a, let-7f and let-7b are predicted based on the genes from mirecords database. miR-181b, miR-181d and miR-605 are predicted based on the genes from miRanda database.

Six miRNAs (miRNA181-b, miRNA-181d, miRNA-605, has-let-7a, has-let-7b, and has-let-7f) were predicted to be related to stroke based on the targetgenes network (Fig. 5). The six predicted miRNAs were associated with 34 genes, 16 of which were the targetgenes of miR-124, such as VIM, KANK1, PRKAG2, and ARPC1B. Five were the targetgenes of let-7 (ESM1, VIM, CDK6, NRAS, and KRAS), 3 of which (ESM1, VIM, and CDK6) were correlated with stroke based on published literatures.

The top 5 most significant functions of let-7 targetgenes according to P value were protein phosphorylation, circulatory system development, multicellular organismal development, cardiovascular system development, and negative regulation of biological process.

### Validation of representative genes and miRNAs

**Figure 6:**
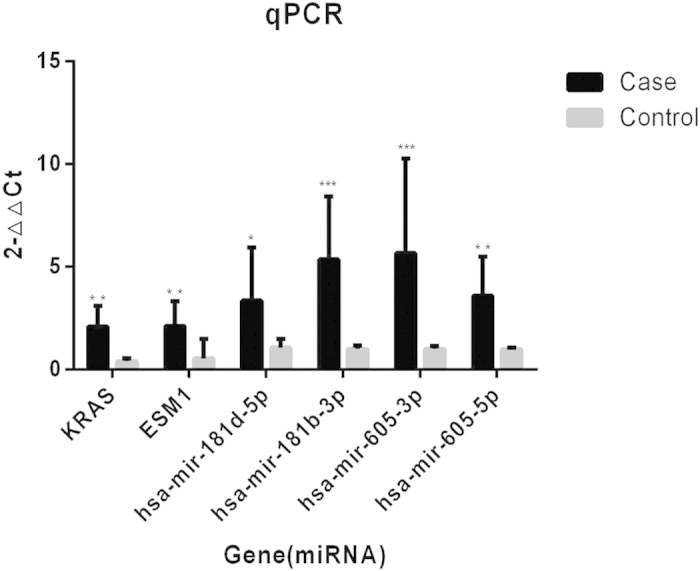
The expression of miRNAs and targetgenes. ESM1, KRAS, miR-181d, miR-181b-3p, miR-605-3p and miR-605-5p were highly expressed in the blood of stroke patients compare to healthy people. *P < 0.05; **P < 0.01; ***P < 0.001.

Next, we examined the expression profiles of predicted miRNAs and their targetgenes by employing qRT-PCR to several stroke patients and healthy people. Finally, ESM1, KRAS, miR-181d, miR-181b-3p, miR-605-3p and miR-605-5p were highly expressed in the blood of stroke patients compared to healthy people (Fig. 6).

## Discussion

In this study, based on the stroke-related miRNAs obtained from the HMDD, we identified miRNA targetgenes, constructed a miRNA-gene functional network to identify important biological processes and predict miRNAs by computational methods. Our findings are partially supported by previous studies.

Our study demonstrated that miR-124 was the most important regulator. A previous study also found that miR-124 was closely related to stroke, which could be used to monitor ischemia-related brain injury starting at 8h and peaking at 24 h after occlusion^8^. Another study indicated that miR-124 regulated Ku70 expression and was correlated with neuronal death induced by ischemia/reperfusion^9^. Moreover, miR-124a was also found to mediate stroke-induced neurogenesis by targeting the JAG-Notch signaling pathway^10^.

Regarded as the effectors of mi-RNA, targetgenes such as SP1, AR, and EGFR had the largest number of edges in the network, indicating that they were closely associated with other genes and played significant roles in stroke. Meanwhile, MAPK signaling pathway was the most important pathway. Our results showed that targetgene SP1 of miR-124 activated EGFR of miR-133a by MAPK cascade. The expression and activation of EGFR and the degree of vessel wall collagen deposition may have protective effect for patients at risk of both ischemic and hemorrhagic strokes^11^. SP1 regulates the ETBR mediated vasoconstriction in focal cerebral ischemia via MEK-ERK signaling. It has been shown that EGFR is involved in two independent downstream signaling pathways, EGFR/DCN/ERK1/2 and EGFR/DCN/ phosphoinositide-3 kinase/AKT, which may mediate the up-regulation and activation of SP1 in mouse cerebral endothelial cells^12^. Inhibition of the MAPK cascade via cytokine suppressive anti-inflammatory drugs, which could block p38 MAPK and hence the production of interleukin-1 and tumor necrosis factor-alpha, is a promising new opportunity for stroke^13^. Though there is a lack of evidence on the correlation between SP1 and EGFR via MAPK cascade in stroke in published literatures, a proper inference could be made in our study.

Many important biological processes were revealed in our study, such as cell death, programmed cell death, MAPK signaling pathway, which are consistent with the findings of previous studies^14^. There were 5 phosphate-related processes in the unique biological processes of module 8, accounting for 17.24%. Following ischemia, alterations occur in both cerebral blood flow and metabolism, which may reflect a relative inhibition of ATP production by metabolic regulators such as ADP on oxidative phosphorylation^15^. Brain injury during stroke results in oxidative stress and the release of factors that include nicotinic acid adenine dinucleotide phosphate^16^. A prior study showed that compared with vehicle control, TAK-242 significantly reduced cerebral infarction, improved neurologic function, and inhibited the phosphorylation of downstream protein kinases in TLR4 signaling pathway^17^.

Additionally, based on the targetgenes network, we predicted the potential miRNAs associated with stroke. let-7, the first known human miRNA, plays a critical role in maintaining vascular health. let-7f was one of the 11 miRNA downloaded from the HMDD. It did not involve in the network construction due to the absence of overlapping genes. However, it was predicted by the other targetgenes of 7 other miRNAs. let-7f is preferentially expressed in microglia in the ischemic hemisphere and antagomir to let-7f promotes neuroprotection in an ischemic stroke model^18^. Circulating let-7b is lower in patients with large-vessel atherosclerosis than healthy volunteers, whereas circulating let-7b has a higher level in patients with other kinds of ischemic stroke until 24 weeks^19^. let-7a expression is downregulated during acute and late phase in rat brain subjected to permanent middle cerebral artery occlusion (MCAO)^20^. Accumulating evidence supports that the human miR-181 family, which constitutes of four members (miR-181a, miR-181b, miR-181c, and miR-181d), plays an important role in stroke^21^,^22^,^23^. The downregulated miR-181b induces neuroprotection against ischemic injury through negatively regulating HSPA5 and UCHL1 protein levels, providing a potential therapeutic target for ischemic stroke^24^.

**Figure 7:**
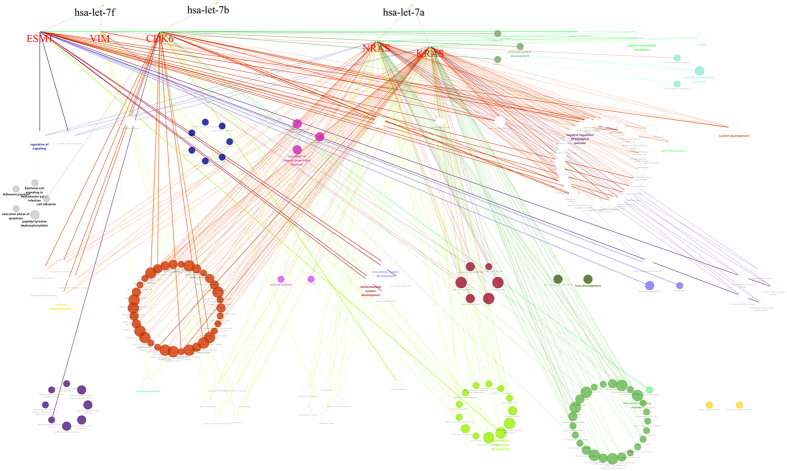
Three predictive miRNAs and biological processes of their targetgenes. ESM1 and vim are the targetgenes of let-7f, CDK6 is the target gene of let-7b, NRAS and KRAS are the targetgenes of let-7a.

Targets and biological processes of the predicted miRNAs were also identified (Fig. 7). Vim and ESM1 are the targets of let-7f; vim is correlated to cell death, regulation of signaling, and signaling transduction. A global gene expression in pre- and post-conditioning-derived neuroprotection in cortical neurons following OGD revealed that vim was upregulated in the neuroprotective cluster genes, and the pathway analysis demonstrated that cell death and signal transduction pathways played a role in neuroprotection^25^. ESM1 was found to be related to cardiovascular system development, circulatory system development, and vasculature development. Cerebral edema attributable to ischemic stroke-induced vascular permeability was reduced by 50% in the absence of ESM1^26^. CDK6 is the target of let-7b. In BV2 microglial cells, it was upregulated in brain of middle cerebral artery occlusion mice *in vitro*^27^.

The targetgenes of miR-181b, such as DDIT4 and Kank1, were also found to be related to stroke. The immunoreactivity and protein levels of DDIT4 were increased in the ischemic gerbil hippocampus proper (CA1 region) at an early time after ischemic damage and the increased DDIT4 expression may be closely related to the delayed neuronal death of the CA1 pyramidal neurons following transient global cerebral ischemia^28^. Kank1 might be involved in cytogenetic behavioral and cerebral ischemia^29^.

## Conclusion

Our study is the latest research on miRNA-genes network in stroke. We not only revealed the widespread biological processes regulated by targetgenes, but also demonstrated the unbalanced impacts of different miRNAs in the network. In our study, miR-605 and miR-181d were predicted to be related to stroke for the first time and we validated the prediction, which were highly expressed in the blood of stroke patients. This may provide potential targets for the diagnosis or treatment of stroke.

## Methods

### Stroke-related miRNAs and targetgenes prediction

Stroke-related miRNAs were obtained from the Human MicroRNA Disease Database (HMDD) (http://cmbi.bjmu.edu.cn/hmdd), a collection of experimentally supported human miRNA and disease associations. miRNAs in the HMDD related to stroke, cerebral hemorrhage, cerebral ischemia, and cerebral infarction were incorporated in our study.

Targetgenes were generated from three independent sources: Targetscan Human 6.2 (http://www.targetscan.org/), miRDB (http://www.mirdb.org/miRDB/) and miRTarBase (http://mirtarbase.mbc.nctu.edu.tw/). We entered the stroke-related miRNAs into the three databases one by one and selected species as “human”. Targetgenes of miRNAs predicted by all three databases were then considered for further analysis.

### Network construction

We imported targetgenes into ClueGO, a Cytoscape plug-in that visualizes the non-redundant biological terms for large clusters of genes in a functionally modulated network.

Kappa score was used to create the network and the functional modules. It was applied to show the relationships between the GO terms based on their overlapping genes. Since the term-tmerm matrix was of categorical origin, kappa statistic was found to be the most suitable method. Initially, a term-gene matrix containing the selected terms and their associated genes was created. Based on this, a term-term similarity matrix was calculated using kappa statistics to determine the association strength between the terms (kappa score)^30^. A term or gene can be included in several functional modules. High score = visualize in the network only the connections between close related terms, with very similar associated genes. Low score = allow to visualize in the network the connections between less related terms. Our study applied a medium score, kappa score = 0.5 (Other parameters: GO tree interval = 3 min level, 8 max level. GO term/pathway selection = 3 min genes, 3% genes.)

### GO functional enrichment analysis

We used the latest precompiled annotation files including STRING, REACTOME, and KEGG for the annotation and pathway of targetgenes. Hypergeometric distribution tests were performed to calculate the significance of each term or group, equivalent to a classical Fisher’s exact test^31^. Bonferroni step-down was used for pV correction. The most representative GO term in a functional module was used to name the module, and it can be considered having the most significant P value. Then, the network was enriched, and all the interactions such as activation, suppression, connection between genes and terms can be reflected by publicly available information from STRING^32^.

### Network analysis

Cytoscape was used to visualize the disease-related networks and analyze the network properties. Network parameters such as the clustering coefficient, network diameter, network centralization, and network radius were determined.

### miRNA prediction

Based on the targetgenes network, we enriched the network with miRNAs prediction file by CluepediaV1.1.5 (edge score = 0.6, threshold = 3), and identified the predicted miRNAs that were most related to the initial targetgenes network.

### RNA extraction and quantitative polymerase chain reaction

Total RNA was isolated from five healthy people and five stroke patients blood samples using the Trizol kit (Life Technologies, US). The qRT-PCR was performed on the StepOnePlus™ Real-Time PCR Systems (ABI, US). The first strand of cDNA was synthesized with 5X FS buffer, DTT, RNase OUT and SuperScript^®^ II Reverse Transcriptase (Life Technologies, US), and corresponding genes were amplified by ABI 9700 (US). The primers used for qRT-PCR were obtained from Life Technologies (Supplementary Table 5).

The StepOnePlus™ Real-Time PCR Systems (ABI) was used for real-time PCR instruments. Reactions were prepared in a total volume of 20 μL containing 10 μL of SYBR Green master mix (Roche Diagnostics, Germany), 0.8 μL of each 10 μM primer, 2 μL cDNA, and 6 μL nuclease-free sterile water. All standards and samples were run in triplicate on 96-well reaction plates. The cycle conditions were set as follows: start with 1 min template denaturation at 95 °C, 40 cycles of denaturation at 95 °C for 5 s, 60 °C for 30 s, 72 °C for 30 s, and elongation at 72 °C for 10 min. This cycle was followed by a melting curve analysis, baseline and cycle threshold values (Ct values) were automatically determined for all plates using StepOne Software v2.0 Software.

### Data analysis

PCR amplification efficiency was calculated using stepone plus V2.0 software. Each reaction was performed in triplicate to check technical consistency and the average of their 2^−△△Ct^ was then used in the analyses. The t test statistic was used for comparison of the genes and miRNAs expression between the control and patient groups (significance level = 0.05). Data was analyzed using SPSS 21.0 software (SPSS Inc, USA).

## Additional Information

**How to cite this article**: Yuan, Y. *et al*. Crosstalk between miRNAs and their regulated genes network in stroke. *Sci. Rep.* **6**, 20429; doi: 10.1038/srep20429 (2016).

## Supplementary Material

Supplementary Information

## Figures and Tables

**Table 1:** Top 10 KEGG pathways and top 10 GO processes.

Top 10 KEGG pathways according to P value			Top 10 GO processes according to P value		
KEGG:04010	MAPK signaling pathway	[Module 6]	GO:0048731	phosphate-containing compound metabolic process	[Module10, Module8]
KEGG:04810	Regulation of actin cytoskeleton	[None]	GO:0007275	multicellular organismal development	[Module10, Module8]
KEGG:04068	FoxO signaling pathway	[Module 6]	GO:0048519	negative regulation of biological process	[Module11]
KEGG:05223	Non-small cell lung cancer	[Module 6]	GO:0006796	system development	[Module7]
KEGG:05220	Chronic myeloid leukemia	[Module 7]	GO:0048523	negative regulation of cellular process	[None]
KEGG:05200	Pathways in cancer	[Module 6]	GO:0030154	cell differentiation	[Module10, Module8]
KEGG:05120	Epithelial cell signaling in Helicobacter pylori infection	[None]	GO:0006793	phosphorus metabolic process	[Module7]
KEGG:05166	HTLV-I infection	[None]	GO:0048869	cellular developmental process	[Module10, Module8]
KEGG:05219	Bladder cancer	[Module 8, Module 7]	GO:0048513	organ development	[Module10]
KEGG:04151	PI3K-Akt signaling pathway	[None]	GO:0009893	positive regulation of metabolic process	[Module11, Module7]

**Table 2:** Number of targetgenes of miR-124 in the top 3 KEGG pathways and GO processes.

KEGG pathways	Associated genes	Targetgenes of miR-124	GO processes	Associated genes	Targetgenes of miR-124
MAPK signaling pathway	20	8	Phosphate-containing compound metabolic process	130	47
Regulation of actin cytoskeleton	19	6	Multicellular organismal development	172	55
FoxO signaling pathway	17	5	Negative regulation of biological process	147	46
