# Supplementary material for: Crosstalk between miRNAs and their regulated genes network in stroke
Source: Sci Rep. 2016 Feb 2;6:20429. doi: 10.1038/srep20429 (PMC4735861; doi:10.1038/srep20429)
Supplement: Supplementary Information [file srep20429-s1.doc]

**Crosstalk between miRNAs and their regulated genes network in stroke**

Ye Yuan^1^, Ruixia Kang^2^, YaNan Yu ^2^, Jun Liu^2^, YingYing Zhang^2^, ChunFeng Shen^3^, Jie Wang ^4^, Ping Wu^4^, ChunTi Shen^3*^, Zhong Wang^2*^

supplementary table 1

Mi-RNAs and their targetgenes

| miRNA | TARGETGENES | NUMBER |
| --- | --- | --- |
| hsa-mir-122 | ALDOA, ANKRD13C, C18orf32, CCNG1, CLIC4, DUSP2, FUNDC2, G6PC3, GNPDA2, GYS1, HECTD3, NPEPPS, P4HA1, PIP4K2A, PKM, SLC7A1 | 16 |
| hsa-mir-124 | ABHD5, ACAA2, ACADVL, AHR, AIF1L, AK2, AKT1S1, AKT3, ALDH9A1, ALG2, AMMECR1, AMMECR1L, ANAPC7, ANKRD27, ANXA11, AP1M2, AR, ARAF, ARFIP1, ARHGEF1, ARMC1, ARPC1B, ASCC2, ATP6V0E1, B4GALT1, C12orf23, C3orf38, CAV1, CBX2, CD151, CD164, CDC14B, CDCA7, CDCP1, CDH2, CDK4, CDK6, CEBPA, CHIC2, CHST14, CHSY1, CLDND1, CMTM4, CNKSR3, COL4A1, CPNE3, CREB3L2, CRTC3, CTDSP1, CTDSP2, CTNND1, CTNS, CYB5A, DACT1, DAPK1, DDX3X, DDX6, DHCR24, DHRS1, DNAJC1, DNM2, DSG2, E2F5, ECI2, EEA1, EGR1, EIF3B, ELK3, ENDOD1, ESYT2, EVI5, EYA4, EZH2, F11R, FAM122B, FAM129B, FAM134B, FAM171A1, FAM177A1, FAM199X, FAM57A, FAM76A, FAR1, FCHO2, FCHSD2, FLOT2, FLRT3, FMNL2, FMR1, FPGS, FRMD6, FRMD8, FSD1L, FXR1, G3BP1, G3BP2, GFPT2, GGA2, GMFB, GNAI2, GNAI3, GNG10, GSN, HADH, HADHA, HECTD2, HIPK3, HTATIP2, IL11, IL6R, INO80C, IQGAP1, ITGA3, ITGB1, ITPRIP, KANK1, KATNA1, KCNK2, KIF26A, KLF6, KLHL24, LAMC1, LCLAT1, LDLRAP1, LHX2, LIMCH1, LIMD2, LITAF, LNX2, LPP, LRIG1, LRRC1, LRRC58, LRRFIP2, MAGT1, MAN2A1, MAP3K13, MAPK14, MEF2A, METAP2, MGAT4A, MKX, MST4, MTMR6, MYH10, MYH9, NAA15, NECAP2, NEK9, NFATC1, NFIA, NID1, NKAP, NME4, NR3C1, NR4A1, NRP1, NSUN2, NUFIP2, OAF, OSBP, OSBPL8, PAM, PAPSS2, PAQR9, PARP16, PARP9, PGM1, PGM2, PGRMC2, PHACTR2, PHF19, PHF6, PI4K2B, PIK3C2A, PLEKHF2, PLEKHM3, PLOD3, PLP2, PLSCR3, PRDM13, PRKAG2, PRPS1, PRRX1, PSKH1, PTBP1, PTPN11, PTPN12, PTPN9, PTPRJ, PTPRZ1, PTTG1IP, PUS3, QKI, QSOX1, RAB11FIP5, RAB27A, RAB34, RANBP10, RARG, RASSF5, RBM24, RBM47, RDH10, RELA, RFFL, RHBDF1, RLIM, RTKN, RHOG, RNPEPL1, RPS6KA4, RRAS, RYK, RYR3, SBNO2, SCAMP2, SDC4, SDF2L1, SEMA5A, SERP1, SERTAD2, SERTAD4, SGK1, SGMS1, SGPL1, SH2B3, SH3PXD2A, SHC1, SHPK, SIX4, SLC15A4, SLC16A1, SLC17A5, SLC22A5, SLC25A30, SLC25A39, SLC29A1, SLC30A7, SLC31A2, SLC35F3, SLC7A1, SLC9A9, SLITRK4, SLITRK5, SMAD5, SMCO4, SNAI2, SNTA1, SNX16, SNX18, SOS2, SP1, SPDL1, SPHK1, SPTY2D1, SSFA2, STK38, STOM, STX10, SYPL1, TADA2B, TAOK1, TARBP1, TEAD1, TFEB, TJP2, TLN1, TMBIM1, TMED1, TMEM104, TMEM109, TMEM184B, TMEM41A, TOM1L1, TOR3A, TP53INP1, TRIB3, TSC22D4, TSEN54, TSKU, TTC7A, TWSG1, TYK2, UHMK1, USP30, USP38, VAMP3, VANGL1, VIM, VPS37C, WASF2, WIPF1, WTAP, ZBTB6, ZCCHC24, ZFP36L2, ZMPSTE24 | 298 |
| hsa-mir-133a | ANKRD28, EGFR, FSCN1, LASP1, MSN, NFAT5, PLEKHA8, RB1CC1, SEC61B, STK4, SUMO1, TAGLN2, VKORC1 | 13 |
| hsa-mir-145 | ABRACL, ADAM17, AP1G1, ARL6IP5, CLINT1, CNOT6L, DENND5B, ERG, FLI1, FSCN1, FZD7, IRS1, ITGB8, MEST, MYO5A, MYO6, NAA30, NEDD9, NRAS, NUFIP2, PBX3, POU5F1, PPP3CA, PSD3, PTP4A2, RLIM, RTKN, SERINC5, SERPINE1, SLC8A3, SRGAP1, STAM, SWAP70, TMEM9B, TPM3, UNC119B, YES1 | 37 |
| hsa-mir-155 | ETS1, FAM135A, FAR1, GNAS, GPM6B, HBP1, HIVEP2, HNRNPA3, INPP5D, JARID2, KRAS, LRRC59, MAP3K10, MAP3K14, MEIS1, MORC3, MYBL1, MYO10, NOVA1, PCDH9, PKN2, RAB5C, RCN2, RREB1, SERTAD2, SMAD2, SPI1, SYPL1, TAB2, TLE4, TOMM20, TP53INP1, TRIM32, TSHZ3, WEE1, WWC1, ZIC3, ZNF236, ZNF652 | 39 |
| hsa-mir-181a | ANKRD13C, ARF6, ATM, ATP8A1, BTBD3, CD69, COPS2, DDIT4, DDX3X, EPHA5, ESM1, FAM122B, FBXO11, FBXO33, FBXO34, GATA6, GIGYF1, H3F3B, HMGB2, KIAA1462, KLF6, MAP2K1, METAP1, MTMR12, NLK, NOTCH2, NR6A1, PCDHA2, PITPNB, PLCL2, PROX1, PUM1, RLF, RNF34, SCD, SIRT1, SRPK2, TGIF2, TM9SF3, TMEM64, TRIM2, YTHDF2, ZBTB41, ZEB2, ZFP36L2, ZNF594 | 46 |
| hsa-mir-298 |  | 0 |
| hsa-mir-362 | E2F1, PTPN1 | 2 |
| hsa-mir-497 |  | 0 |
| hsa-mir-1 |  | 0 |
| hsa-let-7f |  | 0 |

supplementary table 2

Genes and their functions

| NAME | ClusterOrigin | EntrezGeneID | Aliases | Origin | Functions |
| --- | --- | --- | --- | --- | --- |
| ABHD5 | Cluster#1 | 51099 | [CDS, CGI-58, CGI58, IECN2, NCIE2] | Initial Gene From Selection | positive regulation of molecular function | regulation of localization |
| ACAA2 | Cluster#1 | 10449 | [DSAEC] | Initial Gene From Selection | regulation of cellular localization | regulation of transport |
| ACADVL | Cluster#1 | 37 | [ACAD6, LCACD, VLCAD] | Initial Gene From Selection | positive regulation of protein kinase activity | positive regulation of kinase activity |
| ADAM17 | Cluster#1 | 6868 | [ADAM18, CD156B, CSVP, NISBD, TACE] | Initial Gene From Selection | Epithelial cell signaling in Helicobacter pylori infection | positive regulation of protein kinase activity |
| AHR | Cluster#1 | 196 | [bHLHe76] | Initial Gene From Selection | blood vessel development | vasculature development |
| AK2 | Cluster#1 | 204 | [ADK2, AK 2] | Initial Gene From Selection | liver development | hepaticobiliary system development |
| AKT1S1 | Cluster#1 | 84335 | [Lobe, PRAS40] | Initial Gene From Selection | Fc epsilon receptor (FCERI) signaling | regulation of protein kinase activity |
| AKT3 | Cluster#1 | 10000 | [MPPH, PKB-GAMMA, PKBG, PRKBG, RAC-PK-gamma, RAC-gamma, STK-2] | Initial Gene From Selection | Non-small cell lung cancer | Acute myeloid leukemia |
| ALDH9A1 | Cluster#1 | 223 | [ALDH4, ALDH7, ALDH9, E3, TMABADH] | Initial Gene From Selection | liver development | hepaticobiliary system development |
| ALDOA | Cluster#1 | 226 | [ALDA, GSD12, HEL-S-87p] | Initial Gene From Selection | actin cytoskeleton organization | actin filament-based process |
| ALG2 | Cluster#1 | 85365 | [CDGIi, NET38, RP11-13B9.1, hALPG2] | Initial Gene From Selection | peptidyl-amino acid modification | cellular protein modification process | protein modification process |
| ANAPC7 | Cluster#1 | 51434 | [APC7] | Initial Gene From Selection | HTLV-I infection | positive regulation of protein modification process |
| ANKRD13C | Cluster#1 | 81573 | [RP4-677H15.5, dJ677H15.3] | Initial Gene From Selection | cellular protein localization | cellular macromolecule localization |
| ANKRD27 | Cluster#1 | 84079 | [PP12899, VARP] | Initial Gene From Selection | regulation of phosphate metabolic process | regulation of phosphorus metabolic process |
| ANXA11 | Cluster#1 | 311 | [ANX11, CAP50, RP11-369J21.10-010] | Initial Gene From Selection | transport |
| AP1G1 | Cluster#1 | 164 | [ADTG, CLAPG1] | Initial Gene From Selection | regulation of cellular localization | cellular protein localization |
| AP1M2 | Cluster#1 | 10053 | [AP1-mu2, HSMU1B, MU-1B, MU1B, mu2] | Initial Gene From Selection | cellular protein localization | cellular macromolecule localization |
| AR | Cluster#1 | 367 | [AIS, DHTR, HUMARA, HYSP1, KD, NR3C4, RP11-383C12.1, SBMA, SMAX1, TFM] | Initial Gene From Selection | Prostate cancer | negative regulation of extrinsic apoptotic signaling pathway |
| ARAF | Cluster#1 | 369 | [A-RAF, ARAF1, PKS2, RAFA1, RP1-230G1.1] | Initial Gene From Selection | Bladder cancer | Non-small cell lung cancer |
| ARF6 | Cluster#1 | 382 | [] | Initial Gene From Selection | liver development | hepaticobiliary system development |
| ARFIP1 | Cluster#1 | 27236 | [HSU52521] | Initial Gene From Selection | regulation of cellular localization | cellular protein localization |
| ARHGEF1 | Cluster#1 | 9138 | [GEF1, LBCL2, LSC, P115-RHOGEF, SUB1.5] | Initial Gene From Selection | Regulation of actin cytoskeleton | Proteoglycans in cancer |
| ARL6IP5 | Cluster#1 | 10550 | [DERP11, GTRAP3-18, HSPC127, JWA, PRAF3, addicsin, hp22, jmx] | Initial Gene From Selection | regulation of MAPK cascade | MAPK cascade |
| ARMC1 | Cluster#1 | 55156 | [Arcp] | Initial Gene From Selection | transport |
| ARPC1B | Cluster#1 | 10095 | [ARC41, p40-ARC, p41-ARC] | Initial Gene From Selection | Regulation of actin cytoskeleton | actin cytoskeleton organization |
| ASCC2 | Cluster#1 | 84164 | [ASC1p100, SC22CB-11B7.2, p100] | Initial Gene From Selection | regulation of transcription, DNA-templated | regulation of RNA biosynthetic process |
| ATM | Cluster#1 | 472 | [AT1, ATA, ATC, ATD, ATDC, ATE, TEL1, TELO1] | Initial Gene From Selection | FoxO signaling pathway | peptidyl-serine phosphorylation |
| ATP6V0E1 | Cluster#1 | 8992 | [ATP6H, ATP6V0E, M9.2, Vma21, Vma21p] | Initial Gene From Selection | Epithelial cell signaling in Helicobacter pylori infection | insulin receptor signaling pathway |
| ATP8A1 | Cluster#1 | 10396 | [ATPASEII, ATPIA, ATPP2] | Initial Gene From Selection | organic substance transport | transport |
| B4GALT1 | Cluster#1 | 2683 | [B4GAL-T1, CDG2D, GGTB2, GT1, GTB, beta4Gal-T1] | Initial Gene From Selection | angiogenesis | blood vessel morphogenesis |
| BTBD3 | Cluster#1 | 22903 | [RP4-742J24.3, dJ742J24.1] | Initial Gene From Selection | cell morphogenesis involved in neuron differentiation | neuron projection morphogenesis |
| C18orf32 | Cluster#1 | 497661 | [] | Initial Gene From Selection | positive regulation of intracellular signal transduction | positive regulation of signal transduction |
| C3orf38 | Cluster#1 | 285237 | [] | Initial Gene From Selection | apoptotic process | programmed cell death |
| CAV1 | Cluster#1 | 857 | [BSCL3, CGL3, MSTP085, PPH3, VIP21] | Initial Gene From Selection | peptidyl-serine phosphorylation | regulation of extrinsic apoptotic signaling pathway | peptidyl-serine modification |
| CBX2 | Cluster#1 | 84733 | [CDCA6, M33, SRXY5] | Initial Gene From Selection | regulation of transcription from RNA polymerase II promoter | transcription from RNA polymerase II promoter |
| CCNG1 | Cluster#1 | 900 | [CCNG] | Initial Gene From Selection | regulation of protein kinase activity | regulation of kinase activity |
| CD151 | Cluster#1 | 977 | [GP27, MER2, PETA-3, RAPH, SFA1, TSPAN24] | Initial Gene From Selection | cell migration | cell adhesion |
| CD164 | Cluster#1 | 8763 | [MGC-24, MUC-24, RP11-425D10.9, endolyn] | Initial Gene From Selection | hemopoiesis | hematopoietic or lymphoid organ development |
| CD69 | Cluster#1 | 969 | [AIM, BL-AC/P26, CLEC2C, EA1, GP32/28, MLR-3] | Initial Gene From Selection | cellular response to chemical stimulus | signal transduction |
| CDC14B | Cluster#1 | 8555 | [CDC14B3, Cdc14B1, Cdc14B2, RP11-172F4.1, hCDC14B] | Initial Gene From Selection | peptidyl-tyrosine dephosphorylation | positive regulation of protein modification process |
| CDCA7 | Cluster#1 | 83879 | [JPO1] | Initial Gene From Selection | regulation of cell proliferation | apoptotic process |
| CDH2 | Cluster#1 | 1000 | [CD325, CDHN, CDw325, NCAD] | Initial Gene From Selection | Developmental Biology | blood vessel morphogenesis |
| CDK4 | Cluster#1 | 1019 | [CMM3, PSK-J3] | Initial Gene From Selection | Bladder cancer | Non-small cell lung cancer |
| CDK6 | Cluster#1 | 1021 | [PLSTIRE] | Initial Gene From Selection | Non-small cell lung cancer | Glioma |
| CEBPA | Cluster#1 | 1050 | [C/EBP-alpha, CEBP] | Initial Gene From Selection | Acute myeloid leukemia | liver development |
| CHST14 | Cluster#1 | 113189 | [ATCS, D4ST1, EDSMC1, HNK1ST, UNQ1925/PRO4400] | Initial Gene From Selection | cellular biosynthetic process | organic substance biosynthetic process |
| CHSY1 | Cluster#1 | 22856 | [CHSY, CSS1, ChSy-1, TPBS, UNQ756/PRO1487] | Initial Gene From Selection | negative regulation of biological process | cellular biosynthetic process |
| CLIC4 | Cluster#1 | 25932 | [CLIC4L, H1, MTCLIC, huH1, p64H1] | Initial Gene From Selection | angiogenesis | blood vessel morphogenesis |
| CLINT1 | Cluster#1 | 9685 | [CLINT, ENTH, EPN4, EPNR] | Initial Gene From Selection | transport |
| CNKSR3 | Cluster#1 | 154043 | [MAGI1, RP11-486M3.1] | Initial Gene From Selection | peptidyl-serine phosphorylation | peptidyl-serine modification |
| CNOT6L | Cluster#1 | 246175 | [CCR4b] | Initial Gene From Selection | regulation of cell proliferation | regulation of cellular protein metabolic process |
| COL4A1 | Cluster#1 | 1282 | [HANAC, ICH, POREN1, RP11-472K17.2, arresten] | Initial Gene From Selection | Axon guidance | Pathways in cancer |
| COPS2 | Cluster#1 | 9318 | [ALIEN, CSN2, SGN2, TRIP15] | Initial Gene From Selection | generation of neurons | neurogenesis |
| CPNE3 | Cluster#1 | 8895 | [CPN3, PRO1071] | Initial Gene From Selection | protein phosphorylation | phosphorylation |
| CREB3L2 | Cluster#1 | 64764 | [BBF2H7] | Initial Gene From Selection | Prostate cancer | Estrogen signaling pathway |
| CRTC3 | Cluster#1 | 64784 | [TORC-3, TORC3] | Initial Gene From Selection | HTLV-I infection | positive regulation of molecular function |
| CTDSP1 | Cluster#1 | 58190 | [NIF3, NLI-IF, NLIIF, SCP1] | Initial Gene From Selection | regulation of cell differentiation | generation of neurons |
| CTDSP2 | Cluster#1 | 10106 | [OS4, PSR2, SCP2] | Initial Gene From Selection | positive regulation of protein kinase activity | positive regulation of kinase activity |
| CTNND1 | Cluster#1 | 1500 | [CAS, CTNND, P120CAS, P120CTN, p120, p120(CAS), p120(CTN)] | Initial Gene From Selection | Adherens junction | central nervous system development |
| CTNS | Cluster#1 | 1497 | [CTNS-LSB, PQLC4] | Initial Gene From Selection | central nervous system development | nervous system development |
| CYB5A | Cluster#1 | 1528 | [CYB5, MCB5] | Initial Gene From Selection | transport |
| DACT1 | Cluster#1 | 51339 | [DAPPER, DAPPER1, DPR1, FRODO, HDPR1, THYEX3] | Initial Gene From Selection | positive regulation of catenin import into nucleus | regulation of catenin import into nucleus |
| DAPK1 | Cluster#1 | 1612 | [DAPK] | Initial Gene From Selection | negative regulation of extrinsic apoptotic signaling pathway via death domain receptors | Bladder cancer |
| DDIT4 | Cluster#1 | 54541 | [Dig2, REDD-1, REDD1, RP11-442H21.1] | Initial Gene From Selection | peptidyl-serine phosphorylation | peptidyl-serine modification |
| DDX3X | Cluster#1 | 1654 | [DBX, DDX14, DDX3, HLP2] | Initial Gene From Selection | negative regulation of apoptotic signaling pathway | negative regulation of signal transduction |
| DDX6 | Cluster#1 | 1656 | [HLR2, P54, RCK] | Initial Gene From Selection | gene expression | cellular macromolecule metabolic process |
| DENND5B | Cluster#1 | 160518 | [] | Initial Gene From Selection | regulation of intracellular signal transduction | regulation of phosphate metabolic process |
| DHCR24 | Cluster#1 | 1718 | [DCE, Nbla03646, SELADIN1, seladin-1] | Initial Gene From Selection | response to hormone | regulation of apoptotic process |
| DNAJC1 | Cluster#1 | 64215 | [DNAJL1, ERdj1, HTJ1, MTJ1, RP11-399C16.1] | Initial Gene From Selection | regulation of cellular localization | regulation of transport |
| DNM2 | Cluster#1 | 1785 | [CMT2M, CMTDI1, CMTDIB, DI-CMTB, DYN2, DYNII, LCCS5] | Initial Gene From Selection | Axon guidance | Developmental Biology |
| DSG2 | Cluster#1 | 1829 | [ARVC10, ARVD10, CDHF5, CMD1BB, HDGC] | Initial Gene From Selection | actin filament-based movement | execution phase of apoptosis |
| DUSP2 | Cluster#1 | 1844 | [PAC-1, PAC1] | Initial Gene From Selection | peptidyl-tyrosine dephosphorylation | MAPK signaling pathway |
| E2F1 | Cluster#1 | 1869 | [E2F-1, RBAP1, RBBP3, RBP3] | Initial Gene From Selection | enzyme linked receptor protein signaling pathway | positive regulation of gene expression |
| E2F5 | Cluster#1 | 1875 | [E2F-5] | Initial Gene From Selection | Bladder cancer | Non-small cell lung cancer |
| EEA1 | Cluster#1 | 8411 | [MST105, MSTP105, ZFYVE2] | Initial Gene From Selection | transport | cell communication |
| EGFR | Cluster#1 | 1956 | [ERBB, ERBB1, HER1, PIG61, mENA] | Initial Gene From Selection | positive regulation of catenin import into nucleus | SHC1 events in EGFR signaling |
| EGR1 | Cluster#1 | 1958 | [AT225, G0S30, KROX-24, NGFI-A, TIS8, ZIF-268, ZNF225] | Initial Gene From Selection | cellular response to insulin stimulus | HTLV-I infection |
| EIF3B | Cluster#1 | 8662 | [EIF3-ETA, EIF3-P110, EIF3-P116, EIF3S9, PRT1] | Initial Gene From Selection | regulation of cellular protein metabolic process | regulation of protein metabolic process |
| ELK3 | Cluster#1 | 2004 | [ERP, NET, SAP2] | Initial Gene From Selection | angiogenesis | blood vessel morphogenesis |
| EPHA5 | Cluster#1 | 2044 | [CEK7, EHK-1, EHK1, EK7, HEK7, TYRO4] | Initial Gene From Selection | axon guidance | neuron projection guidance | actin cytoskeleton organization |
| ERG | Cluster#1 | 2078 | [erg-3, p55] | Initial Gene From Selection | protein phosphorylation | regulation of transcription from RNA polymerase II promoter |
| ESM1 | Cluster#1 | 11082 | [endocan] | Initial Gene From Selection | angiogenesis | blood vessel morphogenesis |
| ETS1 | Cluster#1 | 2113 | [ETS-1, EWSR2] | Initial Gene From Selection | Dorso-ventral axis formation | execution phase of apoptosis |
| EVI5 | Cluster#1 | 7813 | [NB4S] | Initial Gene From Selection | multicellular organismal development |
| EYA4 | Cluster#1 | 2070 | [CMD1J, DFNA10, RP11-704J17.4] | Initial Gene From Selection | anatomical structure morphogenesis | organ development |
| EZH2 | Cluster#1 | 2146 | [ENX-1, ENX1, EZH1, EZH2b, KMT6, KMT6A, WVS, WVS2] | Initial Gene From Selection | positive regulation of protein kinase activity | positive regulation of kinase activity |
| F11R | Cluster#1 | 50848 | [CD321, JAM, JAM1, JAMA, JCAM, KAT, PAM-1, RP11-544M22.2] | Initial Gene From Selection | Epithelial cell signaling in Helicobacter pylori infection | wound healing |
| FAM129B | Cluster#1 | 64855 | [C9orf88, MEG-3, MINERVA, OC58, RP11-356B19.6, bA356B19.6] | Initial Gene From Selection | regulation of apoptotic process | regulation of programmed cell death |
| FAR1 | Cluster#1 | 84188 | [MLSTD2, SDR10E1, UNQ2423/PRO4981] | Initial Gene From Selection | phosphate-containing compound metabolic process | phosphorus metabolic process |
| FBXO11 | Cluster#1 | 80204 | [FBX11, PRMT9, UBR6, UG063H01, VIT1] | Initial Gene From Selection | peptidyl-amino acid modification | cellular protein modification process | protein modification process |
| FBXO33 | Cluster#1 | 254170 | [BMND12, Fbx33, c14_5247] | Initial Gene From Selection | cellular protein modification process | protein modification process | macromolecule modification |
| FCHO2 | Cluster#1 | 115548 | [] | Initial Gene From Selection | cellular protein localization | cellular macromolecule localization |
| FLI1 | Cluster#1 | 2313 | [EWSR2, SIC-1] | Initial Gene From Selection | regulation of transcription from RNA polymerase II promoter | transcription from RNA polymerase II promoter |
| FLOT2 | Cluster#1 | 2319 | [ECS-1, ECS1, ESA, ESA1, M17S1] | Initial Gene From Selection | Insulin signaling pathway | cell adhesion |
| FLRT3 | Cluster#1 | 23767 | [HH21, UNQ856/PRO1865] | Initial Gene From Selection | axon guidance | neuron projection guidance | axonogenesis |
| FMNL2 | Cluster#1 | 114793 | [FHOD2] | Initial Gene From Selection | actin cytoskeleton organization | actin filament-based process |
| FMR1 | Cluster#1 | 2332 | [FMRP, FRAXA, POF, POF1] | Initial Gene From Selection | central nervous system development | regulation of cellular protein metabolic process |
| FPGS | Cluster#1 | 2356 | [RP11-228B15.1] | Initial Gene From Selection | liver development | hepaticobiliary system development |
| FRMD6 | Cluster#1 | 122786 | [C14orf31, EX1, Willin, c14_5320] | Initial Gene From Selection | actin filament-based movement | actin filament-based process |
| FSCN1 | Cluster#1 | 6624 | [FAN1, HSN, SNL, p55] | Initial Gene From Selection | actin cytoskeleton organization | actin filament-based process |
| FXR1 | Cluster#1 | 8087 | [FXR1P] | Initial Gene From Selection | regulation of cellular protein metabolic process | regulation of protein metabolic process |
| FZD7 | Cluster#1 | 8324 | [FzE3] | Initial Gene From Selection | regulation of catenin import into nucleus | catenin import into nucleus |
| G3BP1 | Cluster#1 | 10146 | [G3BP, HDH-VIII] | Initial Gene From Selection | negative regulation of signal transduction | negative regulation of signaling |
| G3BP2 | Cluster#1 | 9908 | [] | Initial Gene From Selection | regulation of cellular localization | negative regulation of signal transduction |
| G6PC3 | Cluster#1 | 92579 | [SCN4, UGRP] | Initial Gene From Selection | FoxO signaling pathway | Insulin signaling pathway |
| GATA6 | Cluster#1 | 2627 | [] | Initial Gene From Selection | liver development | hepaticobiliary system development |
| GFPT2 | Cluster#1 | 9945 | [GFAT2] | Initial Gene From Selection | peptidyl-amino acid modification | cellular protein modification process | protein modification process |
| GGA2 | Cluster#1 | 23062 | [VEAR] | Initial Gene From Selection | cellular protein localization | cellular macromolecule localization |
| GIGYF1 | Cluster#1 | 64599 | [GYF1, PERQ1, PP3360] | Initial Gene From Selection | enzyme linked receptor protein signaling pathway | cell surface receptor signaling pathway |
| GMFB | Cluster#1 | 2764 | [GMF] | Initial Gene From Selection | regulation of protein kinase activity | regulation of kinase activity |
| GNAI2 | Cluster#1 | 2771 | [GIP, GNAI2B, H_LUCA15.1, H_LUCA16.1] | Initial Gene From Selection | Estrogen signaling pathway | positive regulation of protein kinase activity |
| GNAI3 | Cluster#1 | 2773 | [87U6, ARCND1, RP5-1160K1.2] | Initial Gene From Selection | Estrogen signaling pathway | wound healing |
| GNAS | Cluster#1 | 2778 | [AHO, C20orf45, GNAS1, GPSA, GSA, GSP, NESP, PHP1A, PHP1B, PHP1C, POH, RP4-543J19.4] | Initial Gene From Selection | Estrogen signaling pathway | cellular response to lipid |
| GNG10 | Cluster#1 | 2790 | [] | Initial Gene From Selection | PI3K-Akt signaling pathway | response to hormone |
| GPM6B | Cluster#1 | 2824 | [M6B] | Initial Gene From Selection | actin cytoskeleton organization | actin filament-based process |
| GSN | Cluster#1 | 2934 | [ADF, AGEL, RP11-477J21.1] | Initial Gene From Selection | execution phase of apoptosis | Regulation of actin cytoskeleton |
| GYS1 | Cluster#1 | 2997 | [GSY, GYS] | Initial Gene From Selection | Insulin signaling pathway | PI3K-Akt signaling pathway |
| H3F3B | Cluster#1 | 3021 | [H3.3B] | Initial Gene From Selection | wound healing | response to hormone |
| HADH | Cluster#1 | 3033 | [HAD, HADH1, HADHSC, HCDH, HHF4, MSCHAD, SCHAD] | Initial Gene From Selection | response to insulin | response to hormone |
| HADHA | Cluster#1 | 3030 | [ECHA, GBP, HADH, LCEH, LCHAD, MTPA, TP-ALPHA] | Initial Gene From Selection | response to insulin | response to hormone |
| HBP1 | Cluster#1 | 26959 | [] | Initial Gene From Selection | regulation of transport | regulation of localization |
| HECTD2 | Cluster#1 | 143279 | [RP11-108M11.2] | Initial Gene From Selection | cellular protein modification process | protein modification process | macromolecule modification |
| HECTD3 | Cluster#1 | 79654 | [RP11-69J16.1] | Initial Gene From Selection | cellular protein modification process | protein modification process | macromolecule modification |
| HIPK3 | Cluster#1 | 10114 | [DYRK6, FIST3, PKY, RP1-8L15.1, YAK1] | Initial Gene From Selection | peptidyl-serine phosphorylation | peptidyl-serine modification |
| HIVEP2 | Cluster#1 | 3097 | [HIV-EP2, MBP-2, MIBP1, SHN2, ZAS2, ZNF40B] | Initial Gene From Selection | regulation of transcription, DNA-templated | regulation of RNA biosynthetic process |
| HMGB2 | Cluster#1 | 3148 | [HMG2] | Initial Gene From Selection | negative regulation of extrinsic apoptotic signaling pathway via death domain receptors | negative regulation of extrinsic apoptotic signaling pathway |
| HNRNPA3 | Cluster#1 | 220988 | [2610510D13Rik, D10S102, FBRNP, HNRPA3] | Initial Gene From Selection | gene expression | cellular macromolecule metabolic process |
| HTATIP2 | Cluster#1 | 10553 | [CC3, SDR44U1, TIP30] | Initial Gene From Selection | angiogenesis | blood vessel morphogenesis |
| IL11 | Cluster#1 | 3589 | [AGIF, IL-11] | Initial Gene From Selection | peptidyl-serine phosphorylation | peptidyl-serine modification |
| IL6R | Cluster#1 | 3570 | [CD126, IL-6R-1, IL-6RA, IL6Q, IL6RA, IL6RQ, gp80] | Initial Gene From Selection | PI3K-Akt signaling pathway | positive regulation of protein kinase activity |
| INO80C | Cluster#1 | 125476 | [C18orf37, IES6, hIes6] | Initial Gene From Selection | regulation of transcription, DNA-templated | regulation of RNA biosynthetic process |
| INPP5D | Cluster#1 | 3635 | [SHIP, SHIP-1, SHIP1, SIP-145, hp51CN, p150Ship] | Initial Gene From Selection | hemopoiesis | hematopoietic or lymphoid organ development |
| IQGAP1 | Cluster#1 | 8826 | [HUMORFA01, SAR1, p195] | Initial Gene From Selection | Adherens junction | Regulation of actin cytoskeleton |
| IRS1 | Cluster#1 | 3667 | [HIRS-1] | Initial Gene From Selection | FoxO signaling pathway | Insulin signaling pathway |
| ITGA3 | Cluster#1 | 3675 | [CD49C, GAP-B3, GAPB3, ILNEB, MSK18, VCA-2, VL3A, VLA3a] | Initial Gene From Selection | Regulation of actin cytoskeleton | Pathways in cancer |
| ITGB1 | Cluster#1 | 3688 | [CD29, FNRB, GPIIA, MDF2, MSK12, RP11-479G22.2, VLA-BETA, VLAB] | Initial Gene From Selection | Regulation of actin cytoskeleton | Proteoglycans in cancer |
| ITGB8 | Cluster#1 | 3696 | [] | Initial Gene From Selection | Regulation of actin cytoskeleton | PI3K-Akt signaling pathway |
| ITPRIP | Cluster#1 | 85450 | [DANGER, KIAA1754, RP11-127L20.4, bA127L20, bA127L20.2] | Initial Gene From Selection | negative regulation of extrinsic apoptotic signaling pathway via death domain receptors | negative regulation of extrinsic apoptotic signaling pathway |
| JARID2 | Cluster#1 | 3720 | [JMJ] | Initial Gene From Selection | liver development | hepaticobiliary system development |
| KANK1 | Cluster#1 | 23189 | [ANKRD15, CPSQ2, KANK, RP11-130C19.4] | Initial Gene From Selection | positive regulation of catenin import into nucleus | regulation of catenin import into nucleus |
| KATNA1 | Cluster#1 | 11104 | [] | Initial Gene From Selection | neuron projection development | neuron development |
| KCNK2 | Cluster#1 | 3776 | [K2p2.1, TPKC1, TREK, TREK-1, TREK1, hTREK-1c, hTREK-1e] | Initial Gene From Selection | cell surface receptor signaling pathway | signal transduction |
| KIAA1462 | Cluster#1 | 57608 | [JCAD] | Initial Gene From Selection | cell adhesion |
| KIF26A | Cluster#1 | 26153 | [] | Initial Gene From Selection | wound healing | negative regulation of signal transduction |
| KLF6 | Cluster#1 | 1316 | [BCD1, CBA1, COPEB, CPBP, GBF, PAC1, RP11-184A2.1, ST12, ZF9] | Initial Gene From Selection | hemopoiesis | hematopoietic or lymphoid organ development |
| KRAS | Cluster#1 | 3845 | [C-K-RAS, CFC2, K-RAS2A, K-RAS2B, K-RAS4A, K-RAS4B, KI-RAS, KRAS1, KRAS2, NS, NS3, RASK2] | Initial Gene From Selection | SHC1 events in EGFR signaling | Dorso-ventral axis formation |
| LAMC1 | Cluster#1 | 3915 | [LAMB2, RP11-181K3.1] | Initial Gene From Selection | Axon guidance | Pathways in cancer |
| LASP1 | Cluster#1 | 3927 | [Lasp-1, MLN50] | Initial Gene From Selection | positive regulation of signal transduction | positive regulation of signaling |
| LCLAT1 | Cluster#1 | 253558 | [1AGPAT8, AGPAT8, ALCAT1, HSRG1849, LYCAT, UNQ1849, UNQ1849/PRO3579] | Initial Gene From Selection | phosphate-containing compound metabolic process | phosphorus metabolic process |
| LDLRAP1 | Cluster#1 | 26119 | [ARH, ARH1, ARH2, FHCB1, FHCB2, RP11-70P17.2] | Initial Gene From Selection | regulation of cellular localization | positive regulation of signal transduction |
| LHX2 | Cluster#1 | 9355 | [LH2, hLhx2] | Initial Gene From Selection | axon guidance | neuron projection guidance | axonogenesis |
| LIMCH1 | Cluster#1 | 22998 | [LIMCH1A, LMO7B] | Initial Gene From Selection | actin cytoskeleton organization | actin filament-based process |
| LITAF | Cluster#1 | 9516 | [PIG7, SIMPLE, TP53I7] | Initial Gene From Selection | cellular response to lipid | positive regulation of intracellular signal transduction |
| LPP | Cluster#1 | 4026 | [] | Initial Gene From Selection | cell adhesion |
| LRIG1 | Cluster#1 | 26018 | [LIG-1, LIG1] | Initial Gene From Selection | immune system development | response to oxygen-containing compound |
| LRRFIP2 | Cluster#1 | 9209 | [HUFI-2] | Initial Gene From Selection | cell surface receptor signaling pathway | signal transduction |
| MAGT1 | Cluster#1 | 84061 | [IAP, MRX95, OST3B, PRO0756, RP11-217H1.1, XMEN, bA217H1.1] | Initial Gene From Selection | peptidyl-amino acid modification | cellular protein modification process | protein modification process |
| MAN2A1 | Cluster#1 | 4124 | [AMan II, GOLIM7, MANA2, MANII] | Initial Gene From Selection | liver development | hepaticobiliary system development |
| MAP2K1 | Cluster#1 | 5604 | [CFC3, MAPKK1, MEK1, MKK1, PRKMK1] | Initial Gene From Selection | SHC1 events in EGFR signaling | Dorso-ventral axis formation |
| MAP3K10 | Cluster#1 | 4294 | [MEKK10, MLK2, MST] | Initial Gene From Selection | peptidyl-serine phosphorylation | peptidyl-serine modification |
| MAP3K13 | Cluster#1 | 9175 | [LZK, MEKK13, MLK] | Initial Gene From Selection | MAPK signaling pathway | positive regulation of protein kinase activity |
| MAP3K14 | Cluster#1 | 9020 | [FTDCR1B, HS, HSNIK, NIK] | Initial Gene From Selection | Epithelial cell signaling in Helicobacter pylori infection | T cell receptor signaling pathway |
| MAPK14 | Cluster#1 | 1432 | [CSBP, CSBP1, CSBP2, CSPB1, EXIP, Mxi2, PRKM14, PRKM15, RK, RP1-179N16.5, SAPK2A, p38, p38ALPHA] | Initial Gene From Selection | Epithelial cell signaling in Helicobacter pylori infection | T cell receptor signaling pathway |
| MEF2A | Cluster#1 | 4205 | [ADCAD1, RSRFC4, RSRFC9, mef2] | Initial Gene From Selection | cellular response to lipid | Developmental Biology |
| MEIS1 | Cluster#1 | 4211 | [] | Initial Gene From Selection | hemopoiesis | hematopoietic or lymphoid organ development |
| MEST | Cluster#1 | 4232 | [PEG1] | Initial Gene From Selection | response to oxygen-containing compound | tissue development |
| METAP1 | Cluster#1 | 23173 | [MAP1A, MetAP1A] | Initial Gene From Selection | peptidyl-amino acid modification | regulation of cellular protein metabolic process |
| METAP2 | Cluster#1 | 10988 | [MAP2, MNPEP, p67, p67eIF2] | Initial Gene From Selection | axonogenesis | cell morphogenesis involved in neuron differentiation |
| MGAT4A | Cluster#1 | 11320 | [GNT-IV, GNT-IVA, GnT-4a] | Initial Gene From Selection | peptidyl-amino acid modification | cellular protein modification process | protein modification process |
| MKX | Cluster#1 | 283078 | [C10orf48, IFRX, IRXL1] | Initial Gene From Selection | positive regulation of gene expression | regulation of cell differentiation |
| MORC3 | Cluster#1 | 23515 | [NXP2, ZCW5, ZCWCC3] | Initial Gene From Selection | peptidyl-serine phosphorylation | peptidyl-serine modification |
| MSN | Cluster#1 | 4478 | [HEL70] | Initial Gene From Selection | Regulation of actin cytoskeleton | Proteoglycans in cancer |
| MST4 | Cluster#1 | 51765 | [MASK, RP6-213H19.1] | Initial Gene From Selection | execution phase of apoptosis | signal transduction by phosphorylation |
| MTMR6 | Cluster#1 | 9107 | [RP11-271M24.1] | Initial Gene From Selection | peptidyl-tyrosine dephosphorylation | cellular protein modification process | protein modification process |
| MYBL1 | Cluster#1 | 4603 | [A-MYB, AMYB] | Initial Gene From Selection | HTLV-I infection | positive regulation of gene expression |
| MYH10 | Cluster#1 | 4628 | [NMMHC-IIB, NMMHCB] | Initial Gene From Selection | actin filament-based movement | Regulation of actin cytoskeleton |
| MYH9 | Cluster#1 | 4627 | [BDPLT6, DFNA17, EPSTS, FTNS, MHA, NMHC-II-A, NMMHC-IIA, NMMHCA, RP1-68O2.1] | Initial Gene From Selection | actin filament-based movement | Regulation of actin cytoskeleton |
| MYO10 | Cluster#1 | 4651 | [] | Initial Gene From Selection | Axon guidance | axon guidance | neuron projection guidance |
| MYO5A | Cluster#1 | 4644 | [GS1, MYH12, MYO5, MYR12] | Initial Gene From Selection | actin filament-based movement | cellular response to insulin stimulus |
| MYO6 | Cluster#1 | 4646 | [DFNA22, DFNB37, RP3-472A9.1] | Initial Gene From Selection | actin filament-based movement | actin filament-based process |
| NAA15 | Cluster#1 | 80155 | [Ga19, NARG1, NAT1P, NATH, TBDN, TBDN100] | Initial Gene From Selection | angiogenesis | blood vessel morphogenesis |
| NECAP2 | Cluster#1 | 55707 | [] | Initial Gene From Selection | protein localization | organic substance transport |
| NEDD9 | Cluster#1 | 4739 | [CAS-L, CAS2, CASL, CASS2, HEF1, RP1-49G10.1] | Initial Gene From Selection | actin cytoskeleton organization | actin filament-based process |
| NFAT5 | Cluster#1 | 10725 | [NF-AT5, NFATL1, NFATZ, OREBP, RP11-311C24.1, TONEBP] | Initial Gene From Selection | positive regulation of gene expression | regulation of intracellular signal transduction |
| NFATC1 | Cluster#1 | 4772 | [NF-ATC, NFAT2, NFATc] | Initial Gene From Selection | T cell receptor signaling pathway | Fc epsilon receptor (FCERI) signaling |
| NFIA | Cluster#1 | 4774 | [CTF, NF-I/A, NF1-A, NFI-A, NFI-L, RP5-902P15.1] | Initial Gene From Selection | positive regulation of gene expression | regulation of transcription from RNA polymerase II promoter |
| NID1 | Cluster#1 | 4811 | [NID] | Initial Gene From Selection | cell adhesion | organ development |
| NKAP | Cluster#1 | 79576 | [] | Initial Gene From Selection | hemopoiesis | hematopoietic or lymphoid organ development |
| NLK | Cluster#1 | 51701 | [] | Initial Gene From Selection | Adherens junction | FoxO signaling pathway |
| NME4 | Cluster#1 | 4833 | [NDPK-D, NM23H4, Z97634.4-011, nm23-H4] | Initial Gene From Selection | phosphate-containing compound metabolic process | phosphorus metabolic process |
| NOTCH2 | Cluster#1 | 4853 | [AGS2, HJCYS, hN2] | Initial Gene From Selection | Dorso-ventral axis formation | blood vessel development |
| NOVA1 | Cluster#1 | 4857 | [Nova-1] | Initial Gene From Selection | gene expression | cell communication |
| NPEPPS | Cluster#1 | 9520 | [AAP-S, MP100, PSA] | Initial Gene From Selection | cellular response to chemical stimulus | cellular protein modification process | protein modification process |
| NR3C1 | Cluster#1 | 2908 | [GCCR, GCR, GR, GRL] | Initial Gene From Selection | epithelium development | regulation of apoptotic process |
| NR4A1 | Cluster#1 | 3164 | [GFRP1, HMR, N10, NAK-1, NGFIB, NP10, NUR77, TR3] | Initial Gene From Selection | Fc epsilon receptor (FCERI) signaling | MAPK signaling pathway |
| NR6A1 | Cluster#1 | 2649 | [CT150, GCNF, GCNF1, NR61, RTR, hGCNF, hRTR] | Initial Gene From Selection | regulation of transcription from RNA polymerase II promoter | transcription from RNA polymerase II promoter |
| NRAS | Cluster#1 | 4893 | [ALPS4, N-ras, NRAS1, NS6, RP5-1000E10.2] | Initial Gene From Selection | SHC1 events in EGFR signaling | Bladder cancer |
| NRP1 | Cluster#1 | 8829 | [BDCA4, CD304, NP1, NRP, RP11-342D11.1, VEGF165R] | Initial Gene From Selection | negative regulation of extrinsic apoptotic signaling pathway | regulation of extrinsic apoptotic signaling pathway |
| NSUN2 | Cluster#1 | 54888 | [MISU, MRT5, SAKI, TRM4] | Initial Gene From Selection | macromolecule modification | gene expression |
| OSBP | Cluster#1 | 5007 | [OSBP1] | Initial Gene From Selection | organic substance transport | transport |
| OSBPL8 | Cluster#1 | 114882 | [MST120, MSTP120, ORP8, OSBP10] | Initial Gene From Selection | insulin receptor signaling pathway | cellular response to insulin stimulus |
| P4HA1 | Cluster#1 | 5033 | [P4HA, RP11-344N10.1] | Initial Gene From Selection | peptidyl-amino acid modification | cellular protein modification process | protein modification process |
| PAM | Cluster#1 | 5066 | [PAL, PHM] | Initial Gene From Selection | actin cytoskeleton organization | actin filament-based process |
| PAPSS2 | Cluster#1 | 9060 | [ATPSK2, BCYM4, RP11-77F13.2, SK2] | Initial Gene From Selection | wound healing | cellular response to chemical stimulus |
| PARP16 | Cluster#1 | 54956 | [ARTD15, C15orf30, pART15] | Initial Gene From Selection | positive regulation of protein kinase activity | positive regulation of kinase activity |
| PARP9 | Cluster#1 | 83666 | [ARTD9, BAL, BAL1, MGC:7868] | Initial Gene From Selection | cell migration | cell motility |
| PBX3 | Cluster#1 | 5090 | [RP11-336P12.1] | Initial Gene From Selection | central nervous system development | neuron development |
| PCDH9 | Cluster#1 | 5101 | [RP11-335P18.3] | Initial Gene From Selection | central nervous system development | cell adhesion |
| PCDHA2 | Cluster#1 | 56146 | [PCDH-ALPHA2] | Initial Gene From Selection | cell adhesion | nervous system development |
| PGM1 | Cluster#1 | 5236 | [CDG1T, GSD14] | Initial Gene From Selection | cellular biosynthetic process | organic substance biosynthetic process |
| PGM2 | Cluster#1 | 55276 | [MSTP006] | Initial Gene From Selection | phosphate-containing compound metabolic process | phosphorus metabolic process |
| PGRMC2 | Cluster#1 | 10424 | [DG6, PMBP] | Initial Gene From Selection | cellular response to lipid | response to hormone |
| PHF19 | Cluster#1 | 26147 | [MTF2L1, PCL3, RP11-27I1.1, TDRD19B] | Initial Gene From Selection | positive regulation of protein modification process | positive regulation of cellular protein metabolic process |
| PHF6 | Cluster#1 | 84295 | [AC004383.6, BFLS, BORJ, CENP-31] | Initial Gene From Selection | regulation of transcription, DNA-templated | regulation of RNA biosynthetic process |
| PI4K2B | Cluster#1 | 55300 | [PI4KIIB, PIK42B] | Initial Gene From Selection | phosphate-containing compound metabolic process | phosphorus metabolic process |
| PIK3C2A | Cluster#1 | 5286 | [CPK, PI3-K-C2(ALPHA), PI3-K-C2A] | Initial Gene From Selection | insulin receptor signaling pathway | cellular response to insulin stimulus |
| PIP4K2A | Cluster#1 | 5305 | [PI5P4KA, PIP5K2A, PIP5KII-alpha, PIP5KIIA, PIPK, RP11-301N24.1] | Initial Gene From Selection | Regulation of actin cytoskeleton | hemopoiesis |
| PITPNB | Cluster#1 | 23760 | [PI-TP-beta, PtdInsTP, RP3-353E16.2, VIB1B] | Initial Gene From Selection | organic substance transport | phosphate-containing compound metabolic process |
| PKM | Cluster#1 | 5315 | [CTHBP, HEL-S-30, OIP3, PK3, PKM2, TCB, THBP1] | Initial Gene From Selection | programmed cell death | cell death |
| PKN2 | Cluster#1 | 5586 | [PAK2, PRK2, PRKCL2, PRO2042, Pak-2, RP5-905H16.1] | Initial Gene From Selection | execution phase of apoptosis | peptidyl-serine phosphorylation | regulation of extrinsic apoptotic signaling pathway |
| PLCL2 | Cluster#1 | 23228 | [PLCE2] | Initial Gene From Selection | intracellular signal transduction | signal transduction |
| PLEKHA8 | Cluster#1 | 84725 | [FAPP2] | Initial Gene From Selection | protein localization | organic substance transport |
| PLEKHF2 | Cluster#1 | 79666 | [EAPF, PHAFIN2, ZFYVE18] | Initial Gene From Selection | protein localization | organic substance transport |
| PLEKHM3 | Cluster#1 | 389072 | [DAPR, PLEKHM1L] | Initial Gene From Selection | intracellular signal transduction | signal transduction |
| PLOD3 | Cluster#1 | 8985 | [LH3] | Initial Gene From Selection | response to hormone | cell morphogenesis involved in differentiation |
| PLP2 | Cluster#1 | 5355 | [A4, A4LSB] | Initial Gene From Selection | cellular response to organic substance | cellular response to chemical stimulus |
| PLSCR3 | Cluster#1 | 57048 | [] | Initial Gene From Selection | cellular response to lipid | cellular response to oxygen-containing compound |
| POU5F1 | Cluster#1 | 5460 | [DADB-104B20.2, OCT3, OCT4, OTF-3, OTF3, OTF4, Oct-3, Oct-4] | Initial Gene From Selection | positive regulation of catenin import into nucleus | regulation of catenin import into nucleus |
| PPP3CA | Cluster#1 | 5530 | [CALN, CALNA, CALNA1, CCN1, CNA1, PPP2B] | Initial Gene From Selection | T cell receptor signaling pathway | Fc epsilon receptor (FCERI) signaling |
| PRDM13 | Cluster#1 | 59336 | [MU-MB-20.220, PFM10, RP4-626B19.1] | Initial Gene From Selection | neurogenesis | nervous system development |
| PRKAG2 | Cluster#1 | 51422 | [AAKG, AAKG2, CMH6, H91620p, WPWS] | Initial Gene From Selection | FoxO signaling pathway | Insulin signaling pathway |
| PROX1 | Cluster#1 | 5629 | [] | Initial Gene From Selection | liver development | hepaticobiliary system development |
| PRPS1 | Cluster#1 | 5631 | [ARTS, CMTX5, DFN2, DFNX1, PPRibP, PRS-I, PRSI, RP11-540N4.1] | Initial Gene From Selection | nervous system development | organ development |
| PRRX1 | Cluster#1 | 5396 | [AGOTC, PHOX1, PMX1, PRX-1, PRX1] | Initial Gene From Selection | blood vessel morphogenesis | blood vessel development |
| PSD3 | Cluster#1 | 23362 | [EFA6R, HCA67] | Initial Gene From Selection | generation of neurons | neurogenesis |
| PTBP1 | Cluster#1 | 5725 | [HNRNP-I, HNRNPI, HNRPI, PTB, PTB-1, PTB-T, PTB2, PTB3, PTB4, pPTB] | Initial Gene From Selection | regulation of cell differentiation | cell differentiation |
| PTP4A2 | Cluster#1 | 8073 | [BM-008, HH13, HH7-2, HU-PP-1, OV-1, PRL-2, PRL2, PTP4A, PTPCAAX2, ptp-IV1a, ptp-IV1b] | Initial Gene From Selection | peptidyl-tyrosine dephosphorylation | cellular protein modification process | protein modification process |
| PTPN1 | Cluster#1 | 5770 | [PTP1B] | Initial Gene From Selection | peptidyl-tyrosine dephosphorylation | Adherens junction |
| PTPN11 | Cluster#1 | 5781 | [BPTP3, CFC, NS1, PTP-1D, PTP2C, SH-PTP2, SH-PTP3, SHP2] | Initial Gene From Selection | peptidyl-tyrosine dephosphorylation | Epithelial cell signaling in Helicobacter pylori infection |
| PTPN12 | Cluster#1 | 5782 | [PTP-PEST, PTPG1, tcag7.1075] | Initial Gene From Selection | peptidyl-tyrosine dephosphorylation | cellular protein modification process | protein modification process |
| PTPN9 | Cluster#1 | 5780 | [MEG2, PTPMEG2] | Initial Gene From Selection | peptidyl-tyrosine dephosphorylation | cellular protein modification process | protein modification process |
| PTPRJ | Cluster#1 | 5795 | [CD148, DEP1, HPTPeta, R-PTP-ETA, SCC1] | Initial Gene From Selection | peptidyl-tyrosine dephosphorylation | Adherens junction |
| PTPRZ1 | Cluster#1 | 5803 | [HPTPZ, HPTPzeta, PTP-ZETA, PTP18, PTPRZ, PTPZ, R-PTP-zeta-2, RPTPB, RPTPbeta, phosphacan] | Initial Gene From Selection | peptidyl-tyrosine dephosphorylation | Epithelial cell signaling in Helicobacter pylori infection |
| PTTG1IP | Cluster#1 | 754 | [C21orf1, C21orf3, PBF] | Initial Gene From Selection | cellular protein localization | cellular macromolecule localization |
| PUM1 | Cluster#1 | 9698 | [HSPUM, PUMH, PUMH1, PUML1, RP1-65J11.4] | Initial Gene From Selection | regulation of cellular protein metabolic process | regulation of protein metabolic process |
| PUS3 | Cluster#1 | 83480 | [2610020J05Rik, FKSG32] | Initial Gene From Selection | macromolecule modification | gene expression |
| QKI | Cluster#1 | 9444 | [Hqk, QK, QK1, QK3, hqkI] | Initial Gene From Selection | blood vessel morphogenesis | blood vessel development |
| QSOX1 | Cluster#1 | 5768 | [Q6, QSCN6, RP11-502H18.3] | Initial Gene From Selection | cellular protein metabolic process | cellular macromolecule metabolic process |
| RAB11FIP5 | Cluster#1 | 26056 | [GAF1, RIP11, pp75] | Initial Gene From Selection | cellular response to oxygen-containing compound | regulation of cellular localization |
| RAB27A | Cluster#1 | 5873 | [GS2, HsT18676, RAB27, RAM] | Initial Gene From Selection | wound healing | epithelium development |
| RAB34 | Cluster#1 | 83871 | [RAB39, RAH] | Initial Gene From Selection | cellular protein localization | cellular macromolecule localization |
| RAB5C | Cluster#1 | 5878 | [L1880, RAB5CL, RAB5L, RABL] | Initial Gene From Selection | regulation of transport | regulation of localization |
| RARG | Cluster#1 | 5916 | [NR1B3, RARC] | Initial Gene From Selection | cellular response to lipid | cellular response to oxygen-containing compound |
| RASSF5 | Cluster#1 | 83593 | [Maxp1, NORE1, NORE1A, NORE1B, RAPL, RASSF3, RP11-343H5.1] | Initial Gene From Selection | Non-small cell lung cancer | Pathways in cancer |
| RB1CC1 | Cluster#1 | 9821 | [ATG17, CC1, FIP200] | Initial Gene From Selection | negative regulation of extrinsic apoptotic signaling pathway | liver development |
| RBM24 | Cluster#1 | 221662 | [RNPC6, dJ259A10.1] | Initial Gene From Selection | regulation of cell differentiation | cell differentiation |
| RDH10 | Cluster#1 | 157506 | [SDR16C4, UNQ9375/PRO34191] | Initial Gene From Selection | epithelium development | tissue development |
| RELA | Cluster#1 | 5970 | [NFKB3, p65] | Initial Gene From Selection | Acute myeloid leukemia | Pancreatic cancer |
| RFFL | Cluster#1 | 117584 | [CARP-2, CARP2, FRING, RIFIFYLIN, RNF189, RNF34L] | Initial Gene From Selection | negative regulation of extrinsic apoptotic signaling pathway via death domain receptors | negative regulation of extrinsic apoptotic signaling pathway |
| RHBDF1 | Cluster#1 | 64285 | [C16orf8, Dist1, EGFR-RS, gene-89, gene-90, hDist1] | Initial Gene From Selection | regulation of cellular localization | enzyme linked receptor protein signaling pathway |
| RHOG | Cluster#1 | 391 | [ARHG] | Initial Gene From Selection | Axon guidance | axon guidance | neuron projection guidance |
| RLF | Cluster#1 | 6018 | [RP1-39G22.1, ZN-15L, ZNF292L] | Initial Gene From Selection | positive regulation of gene expression | regulation of transcription from RNA polymerase II promoter |
| RLIM | Cluster#1 | 51132 | [CTD-2530H13.3, NY-REN-43, RNF12] | Initial Gene From Selection | regulation of transcription from RNA polymerase II promoter | transcription from RNA polymerase II promoter |
| RNF34 | Cluster#1 | 80196 | [CARP-1, CARP1, RFI, RIF, RIFF, hRFI] | Initial Gene From Selection | negative regulation of extrinsic apoptotic signaling pathway via death domain receptors | negative regulation of extrinsic apoptotic signaling pathway |
| RNPEPL1 | Cluster#1 | 57140 | [] | Initial Gene From Selection | gene expression | cellular biosynthetic process |
| RPS6KA4 | Cluster#1 | 8986 | [MSK2, RSK-B] | Initial Gene From Selection | peptidyl-serine phosphorylation | peptidyl-serine modification |
| RRAS | Cluster#1 | 6237 | [] | Initial Gene From Selection | Regulation of actin cytoskeleton | Proteoglycans in cancer |
| RREB1 | Cluster#1 | 6239 | [FINB, HNT, LZ321, RP11-69L16.1, RREB-1, Zep-1] | Initial Gene From Selection | positive regulation of gene expression | transcription from RNA polymerase II promoter |
| RTKN | Cluster#1 | 6242 | [] | Initial Gene From Selection | apoptotic process | programmed cell death |
| RYK | Cluster#1 | 6259 | [D3S3195, JTK5, JTK5A, RYK1] | Initial Gene From Selection | axon guidance | neuron projection guidance | regulation of MAPK cascade |
| RYR3 | Cluster#1 | 6263 | [] | Initial Gene From Selection | cellular response to oxygen-containing compound | response to oxygen-containing compound |
| SBNO2 | Cluster#1 | 22904 | [KIAA0963, SNO, STNO] | Initial Gene From Selection | regulation of response to stimulus | regulation of transcription, DNA-templated |
| SCAMP2 | Cluster#1 | 10066 | [] | Initial Gene From Selection | protein localization | organic substance transport |
| SCD | Cluster#1 | 6319 | [FADS5, MSTP008, PRO1933, SCD1, SCDOS] | Initial Gene From Selection | cellular biosynthetic process | organic substance biosynthetic process |
| SDC4 | Cluster#1 | 6385 | [SYND4] | Initial Gene From Selection | Proteoglycans in cancer | positive regulation of protein kinase activity |
| SEC61B | Cluster#1 | 10952 | [] | Initial Gene From Selection | cellular protein localization | cellular macromolecule localization |
| SEMA5A | Cluster#1 | 9037 | [SEMAF, semF] | Initial Gene From Selection | positive regulation of catenin import into nucleus | regulation of catenin import into nucleus |
| SERINC5 | Cluster#1 | 256987 | [C5orf12, TPO1] | Initial Gene From Selection | positive regulation of transferase activity | regulation of transferase activity |
| SERP1 | Cluster#1 | 27230 | [RAMP4] | Initial Gene From Selection | positive regulation of protein kinase activity | positive regulation of kinase activity |
| SERPINE1 | Cluster#1 | 5054 | [PAI, PAI-1, PAI1, PLANH1] | Initial Gene From Selection | negative regulation of extrinsic apoptotic signaling pathway via death domain receptors | negative regulation of extrinsic apoptotic signaling pathway |
| SERTAD2 | Cluster#1 | 9792 | [Sei-2, TRIP-Br2] | Initial Gene From Selection | positive regulation of gene expression | positive regulation of macromolecule metabolic process |
| SGK1 | Cluster#1 | 6446 | [RP1-188K17.1, SGK] | Initial Gene From Selection | FoxO signaling pathway | PI3K-Akt signaling pathway |
| SGMS1 | Cluster#1 | 259230 | [MOB, MOB1, SMS1, TMEM23, hmob33] | Initial Gene From Selection | negative regulation of extrinsic apoptotic signaling pathway | regulation of extrinsic apoptotic signaling pathway |
| SGPL1 | Cluster#1 | 8879 | [S1PL, SPL] | Initial Gene From Selection | execution phase of apoptosis | blood vessel morphogenesis |
| SH2B3 | Cluster#1 | 10019 | [IDDM20, LNK] | Initial Gene From Selection | hemopoiesis | hematopoietic or lymphoid organ development |
| SHC1 | Cluster#1 | 6464 | [RP11-307C12.1, SHC, SHCA] | Initial Gene From Selection | SHC1 events in EGFR signaling | Glioma |
| SHPK | Cluster#1 | 23729 | [CARKL, SHK] | Initial Gene From Selection | cellular response to lipid | cellular response to oxygen-containing compound |
| SIRT1 | Cluster#1 | 23411 | [RP11-57G10.3, SIR2L1] | Initial Gene From Selection | FoxO signaling pathway | insulin receptor signaling pathway |
| SIX4 | Cluster#1 | 51804 | [AREC3] | Initial Gene From Selection | hematopoietic or lymphoid organ development | immune system development |
| SLC15A4 | Cluster#1 | 121260 | [FP12591, PHT1, PTR4] | Initial Gene From Selection | protein localization | organic substance transport |
| SLC16A1 | Cluster#1 | 6566 | [HHF7, MCT, MCT1, RP4-580L15.1] | Initial Gene From Selection | wound healing | cell migration |
| SLC17A5 | Cluster#1 | 26503 | [AST, ISSD, NSD, SD, SIALIN, SIASD, SLD] | Initial Gene From Selection | organic substance transport | transport |
| SLC22A5 | Cluster#1 | 6584 | [CDSP, OCTN2, OCTN2VT] | Initial Gene From Selection | organic substance transport | transport |
| SLC25A30 | Cluster#1 | 253512 | [KMCP1] | Initial Gene From Selection | transport |
| SLC25A39 | Cluster#1 | 51629 | [CGI-69, CGI69] | Initial Gene From Selection | transport | cellular biosynthetic process |
| SLC29A1 | Cluster#1 | 2030 | [ENT1] | Initial Gene From Selection | cellular response to oxygen-containing compound | response to oxygen-containing compound |
| SLC30A7 | Cluster#1 | 148867 | [RP11-30G24.1, ZNT7, ZnT-7, ZnTL2] | Initial Gene From Selection | cellular protein metabolic process | transport |
| SLC31A2 | Cluster#1 | 1318 | [COPT2, CTR2, RP11-9H12.2, hCTR2] | Initial Gene From Selection | transport |
| SLC35F3 | Cluster#1 | 148641 | [] | Initial Gene From Selection | transport |
| SLC7A1 | Cluster#1 | 6541 | [ATRC1, CAT-1, ERR, HCAT1, REC1L, RP11-274A8.1] | Initial Gene From Selection | organic substance transport | transport |
| SLC8A3 | Cluster#1 | 6547 | [NCX3] | Initial Gene From Selection | wound healing | cellular response to oxygen-containing compound |
| SLC9A9 | Cluster#1 | 285195 | [AUTS16, NHE9, Nbla00118] | Initial Gene From Selection | transport |
| SLITRK4 | Cluster#1 | 139065 | [] | Initial Gene From Selection | axonogenesis | cell morphogenesis involved in neuron differentiation |
| SLITRK5 | Cluster#1 | 26050 | [LRRC11, bA364G4.2] | Initial Gene From Selection | axonogenesis | cell morphogenesis involved in neuron differentiation |
| SMAD2 | Cluster#1 | 4087 | [JV18, JV18-1, MADH2, MADR2, hMAD-2, hSMAD2] | Initial Gene From Selection | Pancreatic cancer | Adherens junction |
| SMAD5 | Cluster#1 | 4090 | [DWFC, JV5-1, MADH5] | Initial Gene From Selection | hemopoiesis | hematopoietic or lymphoid organ development |
| SNAI2 | Cluster#1 | 6591 | [SLUG, SLUGH1, SNAIL2, WS2D] | Initial Gene From Selection | regulation of catenin import into nucleus | catenin import into nucleus |
| SNTA1 | Cluster#1 | 6640 | [LQT12, SNT1, TACIP1, dJ1187J4.5] | Initial Gene From Selection | actin filament-based movement | actin filament-based process |
| SNX16 | Cluster#1 | 64089 | [] | Initial Gene From Selection | cellular protein localization | cellular macromolecule localization |
| SNX18 | Cluster#1 | 112574 | [SH3PX2, SH3PXD3B, SNAG1] | Initial Gene From Selection | cellular protein localization | cellular macromolecule localization |
| SOS2 | Cluster#1 | 6655 | [] | Initial Gene From Selection | Dorso-ventral axis formation | Non-small cell lung cancer |
| SP1 | Cluster#1 | 6667 | [] | Initial Gene From Selection | Estrogen signaling pathway | liver development |
| SPDL1 | Cluster#1 | 54908 | [CCDC99] | Initial Gene From Selection | cellular protein localization | cellular macromolecule localization |
| SPHK1 | Cluster#1 | 8877 | [SPHK] | Initial Gene From Selection | angiogenesis | blood vessel morphogenesis |
| SPI1 | Cluster#1 | 6688 | [OF, PU.1, SFPI1, SPI-1, SPI-A, hCG_25181] | Initial Gene From Selection | Acute myeloid leukemia | HTLV-I infection |
| SRGAP1 | Cluster#1 | 57522 | [ARHGAP13] | Initial Gene From Selection | Axon guidance | axon guidance | neuron projection guidance |
| SRPK2 | Cluster#1 | 6733 | [SFRSK2] | Initial Gene From Selection | angiogenesis | blood vessel morphogenesis |
| STAM | Cluster#1 | 8027 | [STAM-1, STAM1] | Initial Gene From Selection | negative regulation of signal transduction | negative regulation of signaling |
| STK38 | Cluster#1 | 11329 | [NDR, NDR1] | Initial Gene From Selection | regulation of MAPK cascade | MAPK cascade |
| STK4 | Cluster#1 | 6789 | [KRS2, MST1, TIIAC, YSK3] | Initial Gene From Selection | Non-small cell lung cancer | FoxO signaling pathway |
| STX10 | Cluster#1 | 8677 | [SYN10, hsyn10] | Initial Gene From Selection | cellular protein localization | cellular macromolecule localization |
| SUMO1 | Cluster#1 | 7341 | [DAP1, GMP1, OFC10, OK/SW-cl.43, PIC1, SENP2, SMT3, SMT3C, SMT3H3, UBL1] | Initial Gene From Selection | positive regulation of protein phosphorylation | positive regulation of phosphorylation |
| SWAP70 | Cluster#1 | 23075 | [HSPC321, SWAP-70] | Initial Gene From Selection | immune system development | system development |
| SYPL1 | Cluster#1 | 6856 | [H-SP1, SYPL] | Initial Gene From Selection | cell communication |
| TAB2 | Cluster#1 | 23118 | [CHTD2, MAP3K7IP2] | Initial Gene From Selection | Fc epsilon receptor (FCERI) signaling | MAPK signaling pathway |
| TADA2B | Cluster#1 | 93624 | [ADA2(beta), ADA2B] | Initial Gene From Selection | regulation of transcription, DNA-templated | regulation of RNA biosynthetic process |
| TAGLN2 | Cluster#1 | 8407 | [HA1756, RP11-48O20.1] | Initial Gene From Selection | epithelium development | tissue development |
| TAOK1 | Cluster#1 | 57551 | [KFC-B, MAP3K16, MARKK, PSK-2, PSK2, TAO1, hKFC-B, hTAOK1] | Initial Gene From Selection | execution phase of apoptosis | MAPK signaling pathway |
| TARBP1 | Cluster#1 | 6894 | [RP5-827C21.5, TRM3, TRP-185, TRP185] | Initial Gene From Selection | regulation of transcription from RNA polymerase II promoter | transcription from RNA polymerase II promoter |
| TEAD1 | Cluster#1 | 7003 | [AA, NTEF-1, REF1, TCF-13, TCF13, TEAD-1, TEF-1] | Initial Gene From Selection | epithelium development | cardiovascular system development | circulatory system development |
| TFEB | Cluster#1 | 7942 | [ALPHATFEB, BHLHE35, RP4-696P19.3, TCFEB] | Initial Gene From Selection | positive regulation of gene expression | regulation of transcription from RNA polymerase II promoter |
| TGIF2 | Cluster#1 | 60436 | [] | Initial Gene From Selection | enzyme linked receptor protein signaling pathway | regulation of transcription from RNA polymerase II promoter |
| TJP2 | Cluster#1 | 9414 | [C9DUPq21.11, DFNA51, DUP9q21.11, RP11-16N10.1, X104, ZO2] | Initial Gene From Selection | execution phase of apoptosis | phosphorylation |
| TLE4 | Cluster#1 | 7091 | [BCE-1, BCE1, E(spI), ESG, ESG4, GRG4, RP11-79D8.3] | Initial Gene From Selection | regulation of transcription from RNA polymerase II promoter | transcription from RNA polymerase II promoter |
| TLN1 | Cluster#1 | 7094 | [ILWEQ, RP11-112J3.1, TLN] | Initial Gene From Selection | HTLV-I infection | Axon guidance |
| TMED1 | Cluster#1 | 11018 | [IL1RL1LG, Il1rl1l, Tp24] | Initial Gene From Selection | protein localization | organic substance transport |
| TMEM109 | Cluster#1 | 79073 | [] | Initial Gene From Selection | regulation of cell death | apoptotic process |
| TMEM64 | Cluster#1 | 169200 | [] | Initial Gene From Selection | hemopoiesis | hematopoietic or lymphoid organ development |
| TMEM9B | Cluster#1 | 56674 | [C11orf15, UNQ712/PRO1375] | Initial Gene From Selection | positive regulation of intracellular signal transduction | positive regulation of signal transduction |
| TOM1L1 | Cluster#1 | 10040 | [OK/KNS-CL.3, SRCASM] | Initial Gene From Selection | positive regulation of protein kinase activity | positive regulation of kinase activity |
| TOMM20 | Cluster#1 | 9804 | [MAS20, MOM19, RP4-597N16.2, TOM20] | Initial Gene From Selection | cellular protein localization | cellular macromolecule localization |
| TOR3A | Cluster#1 | 64222 | [ADIR, ADIR2, RP11-177A2.2] | Initial Gene From Selection | phosphate-containing compound metabolic process | phosphorus metabolic process |
| TP53INP1 | Cluster#1 | 94241 | [SIP, TP53DINP1, TP53INP1A, TP53INP1B, Teap, p53DINP1] | Initial Gene From Selection | HTLV-I infection | cellular response to oxygen-containing compound |
| TPM3 | Cluster#1 | 7170 | [CAPM1, CFTD, HEL-189, HEL-S-82p, NEM1, OK/SW-cl.5, RP11-205M9.1, TM-5, TM3, TM30, TM30nm, TM5, TPMsk3, TRK, hscp30] | Initial Gene From Selection | actin filament-based movement | Pathways in cancer |
| TRIB3 | Cluster#1 | 57761 | [C20orf97, NIPK, RP5-1103G7.7, SINK, SKIP3, TRB3] | Initial Gene From Selection | insulin receptor signaling pathway | Fc epsilon receptor (FCERI) signaling |
| TRIM2 | Cluster#1 | 23321 | [CMT2R, RNF86] | Initial Gene From Selection | regulation of apoptotic process | regulation of programmed cell death |
| TRIM32 | Cluster#1 | 22954 | [BBS11, HT2A, LGMD2H, RP11-67K19.1, TATIP] | Initial Gene From Selection | negative regulation of apoptotic signaling pathway | positive regulation of intracellular signal transduction |
| TSC22D4 | Cluster#1 | 81628 | [THG-1, THG1, TILZ2] | Initial Gene From Selection | regulation of transcription, DNA-templated | regulation of RNA biosynthetic process |
| TSEN54 | Cluster#1 | 283989 | [PCH2A, PCH4, SEN54L, sen54] | Initial Gene From Selection | gene expression | cellular macromolecule metabolic process |
| TSHZ3 | Cluster#1 | 57616 | [TSH3, ZNF537] | Initial Gene From Selection | regulation of cell differentiation | tissue development |
| TSKU | Cluster#1 | 25987 | [E2IG4, LRRC54, TSK, UNQ850/PRO1788] | Initial Gene From Selection | axonogenesis | cell morphogenesis involved in neuron differentiation |
| TTC7A | Cluster#1 | 57217 | [MINAT, TTC7] | Initial Gene From Selection | hemopoiesis | hematopoietic or lymphoid organ development |
| TWSG1 | Cluster#1 | 57045 | [PSEC0250, TSG] | Initial Gene From Selection | hemopoiesis | hematopoietic or lymphoid organ development |
| TYK2 | Cluster#1 | 7297 | [JTK1] | Initial Gene From Selection | peptidyl-amino acid modification | protein phosphorylation |
| UHMK1 | Cluster#1 | 127933 | [KIS, KIST, P-CIP2] | Initial Gene From Selection | peptidyl-serine phosphorylation | peptidyl-serine modification |
| UNC119B | Cluster#1 | 84747 | [POC7B, hCG_27366] | Initial Gene From Selection | cell projection morphogenesis | cell part morphogenesis |
| USP30 | Cluster#1 | 84749 | [] | Initial Gene From Selection | cellular protein modification process | protein modification process | macromolecule modification |
| USP38 | Cluster#1 | 84640 | [HP43.8KD] | Initial Gene From Selection | cellular protein modification process | protein modification process | macromolecule modification |
| VAMP3 | Cluster#1 | 9341 | [CEB] | Initial Gene From Selection | cell morphogenesis involved in differentiation | cell adhesion |
| VANGL1 | Cluster#1 | 81839 | [KITENIN, LPP2, STB2, STBM2] | Initial Gene From Selection | multicellular organismal development |
| VIM | Cluster#1 | 7431 | [CTRCT30, HEL113, RP11-124N14.1] | Initial Gene From Selection | actin filament-based movement | execution phase of apoptosis |
| VKORC1 | Cluster#1 | 79001 | [EDTP308, IMAGE3455200, MST134, MST576, MSTP134, VKCFD2, VKOR] | Initial Gene From Selection | peptidyl-amino acid modification | cellular protein modification process | protein modification process |
| VPS37C | Cluster#1 | 55048 | [] | Initial Gene From Selection | protein localization | organic substance transport |
| WASF2 | Cluster#1 | 10163 | [IMD2, SCAR2, WASF4, WAVE2, dJ393P12.2] | Initial Gene From Selection | Adherens junction | actin filament-based movement |
| WEE1 | Cluster#1 | 7465 | [WEE1A, WEE1hu] | Initial Gene From Selection | neuron projection morphogenesis | wound healing |
| WIPF1 | Cluster#1 | 7456 | [PRPL-2, WASPIP, WIP] | Initial Gene From Selection | actin filament-based movement | actin cytoskeleton organization |
| WTAP | Cluster#1 | 9589 | [RP1-56L9.4] | Initial Gene From Selection | gene expression | cellular macromolecule metabolic process |
| WWC1 | Cluster#1 | 23286 | [HBEBP3, HBEBP36, KIBRA, MEMRYQTL] | Initial Gene From Selection | regulation of MAPK cascade | MAPK cascade |
| YES1 | Cluster#1 | 7525 | [HsT441, P61-YES, Yes, c-yes] | Initial Gene From Selection | Adherens junction | wound healing |
| YTHDF2 | Cluster#1 | 51441 | [HGRG8, NY-REN-2] | Initial Gene From Selection | regulation of gene expression | regulation of macromolecule metabolic process |
| ZBTB41 | Cluster#1 | 360023 | [FRBZ1, RP11-469L3.1, ZNF924] | Initial Gene From Selection | regulation of transcription, DNA-templated | regulation of RNA biosynthetic process |
| ZBTB6 | Cluster#1 | 10773 | [ZID, ZNF482] | Initial Gene From Selection | regulation of transcription, DNA-templated | regulation of RNA biosynthetic process |
| ZEB2 | Cluster#1 | 9839 | [HRIHFB2411, HSPC082, SIP-1, SIP1, SMADIP1, ZFHX1B] | Initial Gene From Selection | positive regulation of protein kinase activity | positive regulation of kinase activity |
| ZFP36L2 | Cluster#1 | 678 | [BRF2, ERF-2, ERF2, RNF162C, TIS11D] | Initial Gene From Selection | hemopoiesis | hematopoietic or lymphoid organ development |
| ZIC3 | Cluster#1 | 7547 | [HTX, HTX1, RP1-137H15.3, VACTERLX, ZNF203] | Initial Gene From Selection | liver development | hepaticobiliary system development |
| ZMPSTE24 | Cluster#1 | 10269 | [FACE-1, FACE1, HGPS, PRO1, STE24, Ste24p] | Initial Gene From Selection | cellular protein metabolic process | gene expression |
| ZNF236 | Cluster#1 | 7776 | [ZNF236A, ZNF236B] | Initial Gene From Selection | cellular response to oxygen-containing compound | response to oxygen-containing compound |
| ZNF594 | Cluster#1 | 84622 | [hCG_1775942] | Initial Gene From Selection | regulation of transcription, DNA-templated | regulation of RNA biosynthetic process |
| ZNF652 | Cluster#1 | 22834 | [] | Initial Gene From Selection | regulation of transcription, DNA-templated | regulation of RNA biosynthetic process |

supplementary table 3

GO Term and GO Modules

| GO ID | GO Term | GO Modules | Nr. Genes | Term PValue |
| --- | --- | --- | --- | --- |
| GO:0000165 | MAPK cascade | [Module7] | 32 | 3.03E-05 |
| GO:0000902 | cell morphogenesis | [Module10, Module8] | 52 | 3.37E-06 |
| GO:0000904 | cell morphogenesis involved in differentiation | [Module10, Module8] | 44 | 3.51E-07 |
| GO:0001525 | angiogenesis | [Module4] | 25 | 1.46E-05 |
| GO:0001568 | blood vessel development | [Module4] | 37 | 2.33E-08 |
| GO:0001889 | liver development | [None] | 14 | 7.23E-07 |
| GO:0001932 | regulation of protein phosphorylation | [Module7] | 48 | 8.72E-07 |
| GO:0001934 | positive regulation of protein phosphorylation | [Module7] | 38 | 7.96E-07 |
| GO:0001944 | vasculature development | [Module4] | 38 | 2.43E-08 |
| GO:0002520 | immune system development | [None] | 37 | 1.16E-05 |
| KEGG:04010 | MAPK signaling pathway | [Module6] | 20 | 3.58E-10 |
| KEGG:04810 | Regulation of actin cytoskeleton | [None] | 19 | 9.53E-09 |
| KEGG:04068 | FoxO signaling pathway | [Module6] | 17 | 1.73E-08 |
| KEGG:05223 | Non-small cell lung cancer | [Module6] | 12 | 5.95E-08 |
| KEGG:05220 | Chronic myeloid leukemia | [Module6] | 12 | 1.36E-07 |
| KEGG:05200 | Pathways in cancer | [Module6] | 25 | 3.02E-07 |
| KEGG:05120 | Epithelial cell signaling in Helicobacter pylori infection | [None] | 9 | 3.14E-07 |
| KEGG:05166 | HTLV-I infection | [None] | 22 | 3.43E-07 |
| KEGG:05219 | Bladder cancer | [Module5, Module6] | 8 | 2.48E-06 |
| KEGG:04151 | PI3K-Akt signaling pathway | [None] | 24 | 2.93E-06 |
| KEGG:05212 | Pancreatic cancer | [Module6] | 10 | 3.29E-06 |
| KEGG:04915 | Estrogen signaling pathway | [Module5, Module6] | 12 | 4.37E-06 |
| KEGG:05221 | Acute myeloid leukemia | [Module6] | 9 | 7.22E-06 |
| KEGG:05205 | Proteoglycans in cancer | [None] | 18 | 7.51E-06 |
| KEGG:05215 | Prostate cancer | [Module6] | 11 | 8.21E-06 |
| KEGG:04520 | Adherens junction | [None] | 10 | 8.37E-06 |
| KEGG:04320 | Dorso-ventral axis formation | [Module5] | 6 | 1.62E-05 |
| KEGG:05120 | Epithelial cell signaling in Helicobacter pylori infection | [None] | 9 | 3.14E-05 |
| KEGG:04660 | T cell receptor signaling pathway | [Module6] | 11 | 3.65E-05 |
| KEGG:05218 | Melanoma | [Module5, Module6] | 9 | 4.46E-05 |
| KEGG:04910 | Insulin signaling pathway | [Module6] | 13 | 3.19E-07 |
| KEGG:05214 | Glioma | [Module5, Module6] | 11 | 3.35E-07 |
| GO:0006357 | regulation of transcription from RNA polymerase II promoter | [Module11] | 62 | 8.16E-06 |
| GO:0006366 | transcription from RNA polymerase II promoter | [Module11] | 66 | 4.09E-05 |
| GO:0006464 | cellular protein modification process | [Module1, Module7] | 109 | 9.90E-08 |
| GO:0006468 | protein phosphorylation | [Module1, Module7] | 68 | 3.62E-10 |
| GO:0006793 | phosphorus metabolic process | [Module7] | 130 | 1.55E-11 |
| GO:0006796 | phosphate-containing compound metabolic process | [Module7] | 130 | 4.81E-12 |
| GO:0006810 | transport | [Module3] | 134 | 1.85E-05 |
| GO:0006915 | apoptotic process | [Module12] | 80 | 1.17E-08 |
| GO:0006928 | cellular component movement | [None] | 77 | 5.35E-09 |
| GO:0007154 | cell communication | [Module2, Module7] | 191 | 7.95E-10 |
| GO:0007155 | cell adhesion | [None] | 47 | 3.55E-05 |
| GO:0007165 | signal transduction | [Module2, Module7] | 175 | 9.42E-10 |
| GO:0007166 | cell surface receptor signaling pathway | [Module7, Module9] | 103 | 1.13E-05 |
| GO:0007167 | enzyme linked receptor protein signaling pathway | [Module7, Module9] | 45 | 3.35E-05 |
| GO:0007275 | multicellular organismal development | [Module10, Module8] | 172 | 2.85E-12 |
| GO:0007399 | nervous system development | [Module10, Module8] | 87 | 2.06E-08 |
| GO:0007409 | axonogenesis | [Module8] | 31 | 3.22E-05 |
| GO:0007411 | axon guidance | [Module8] | 24 | 4.02E-05 |
| GO:0007417 | central nervous system development | [Module8] | 43 | 4.37E-06 |
| GO:0008104 | protein localization | [Module3] | 74 | 1.11E-05 |
| GO:0008219 | cell death | [Module12] | 83 | 2.60E-07 |
| GO:0008286 | insulin receptor signaling pathway | [Module6, Module9] | 15 | 2.34E-05 |
| GO:0009653 | anatomical structure morphogenesis | [Module10, Module8] | 102 | 4.95E-10 |
| GO:0009725 | response to hormone | [Module9] | 40 | 6.42E-06 |
| GO:0009888 | tissue development | [None] | 65 | 6.14E-06 |
| GO:0009889 | regulation of biosynthetic process | [Module13, Module11] | 132 | 7.15E-07 |
| GO:0009893 | positive regulation of metabolic process | [Module11, Module7] | 105 | 1.41E-10 |
| GO:0009966 | regulation of signal transduction | [Module7] | 99 | 2.15E-09 |
| GO:0009967 | positive regulation of signal transduction | [Module7] | 51 | 1.81E-06 |
| GO:0009968 | negative regulation of signal transduction | [Module7] | 45 | 2.08E-06 |
| GO:0010033 | response to organic substance | [Module9] | 96 | 8.45E-08 |
| GO:0010467 | gene expression | [Module13, Module11] | 166 | 4.23E-05 |
| GO:0010468 | regulation of gene expression | [Module11] | 137 | 1.76E-07 |
| GO:0010556 | regulation of macromolecule biosynthetic process | [Module13, Module11] | 127 | 5.72E-07 |
| GO:0010562 | positive regulation of phosphorus metabolic process | [Module11, Module7] | 45 | 8.89E-07 |
| GO:0010604 | positive regulation of macromolecule metabolic process | [Module11, Module7] | 98 | 1.73E-10 |
| GO:0010628 | positive regulation of gene expression | [Module11] | 55 | 1.04E-05 |
| GO:0010646 | regulation of cell communication | [Module7] | 107 | 3.43E-09 |
| GO:0010647 | positive regulation of cell communication | [Module7] | 52 | 3.32E-06 |
| GO:0010648 | negative regulation of cell communication | [Module7] | 46 | 3.91E-06 |
| GO:0010941 | regulation of cell death | [Module12] | 61 | 3.10E-06 |
| GO:0012501 | programmed cell death | [Module12] | 81 | 8.62E-09 |
| REACTOME:12579 | SHC1 events in EGFR signaling | [Module5, Module6] | 5 | 1.82E-05 |
| GO:0016310 | phosphorylation | [Module1, Module7] | 81 | 2.47E-10 |
| GO:0016477 | cell migration | [None] | 55 | 1.45E-08 |
| GO:0018105 | peptidyl-serine phosphorylation | [None] | 15 | 1.56E-05 |
| GO:0018193 | peptidyl-amino acid modification | [None] | 41 | 9.02E-06 |
| GO:0018209 | peptidyl-serine modification | [None] | 15 | 3.46E-05 |
| REACTOME:18266 | Axon guidance | [Module8] | 21 | 2.19E-06 |
| GO:0019219 | regulation of nucleobase-containing compound metabolic process | [Module11] | 132 | 8.08E-06 |
| GO:0019220 | regulation of phosphate metabolic process | [Module7] | 69 | 3.59E-06 |
| GO:0019222 | regulation of metabolic process | [Module13, Module11, Module2] | 189 | 1.01E-08 |
| GO:0022008 | neurogenesis | [Module10, Module8] | 58 | 1.00E-05 |
| GO:0023014 | signal transduction by phosphorylation | [Module7] | 35 | 2.77E-06 |
| GO:0023051 | regulation of signaling | [Module7] | 108 | 1.33E-09 |
| GO:0023056 | positive regulation of signaling | [Module7] | 53 | 1.18E-06 |
| GO:0023057 | negative regulation of signaling | [Module7] | 46 | 3.75E-06 |
| GO:0030029 | actin filament-based process | [None] | 35 | 5.00E-07 |
| GO:0030036 | actin cytoskeleton organization | [None] | 28 | 3.24E-05 |
| GO:0030048 | actin filament-based movement | [None] | 11 | 3.65E-05 |
| GO:0030097 | hemopoiesis | [None] | 33 | 2.30E-05 |
| GO:0030154 | cell differentiation | [Module10, Module8] | 133 | 6.46E-12 |
| GO:0031175 | neuron projection development | [Module8] | 43 | 1.95E-06 |
| GO:0031323 | regulation of cellular metabolic process | [Module13, Module11, Module2] | 173 | 2.74E-08 |
| GO:0031325 | positive regulation of cellular metabolic process | [Module11, Module7] | 97 | 4.25E-09 |
| GO:0031326 | regulation of cellular biosynthetic process | [Module13, Module11] | 131 | 6.37E-07 |
| GO:0031399 | regulation of protein modification process | [Module1, Module11, Module7] | 57 | 1.14E-06 |
| GO:0031401 | positive regulation of protein modification process | [Module11, Module7] | 45 | 7.93E-07 |
| GO:0032268 | regulation of cellular protein metabolic process | [Module1, Module11, Module7] | 71 | 2.40E-08 |
| GO:0032270 | positive regulation of cellular protein metabolic process | [Module11, Module7] | 49 | 5.35E-07 |
| GO:0032868 | response to insulin | [Module9] | 19 | 3.70E-05 |
| GO:0032869 | cellular response to insulin stimulus | [Module9] | 17 | 4.38E-05 |
| GO:0032879 | regulation of localization | [Module3] | 78 | 1.20E-08 |
| GO:0032989 | cellular component morphogenesis | [Module10, Module8] | 52 | 1.77E-05 |
| GO:0032990 | cell part morphogenesis | [Module8] | 39 | 4.38E-05 |
| GO:0033674 | positive regulation of kinase activity | [Module7] | 30 | 2.79E-06 |
| GO:0034613 | cellular protein localization | [Module3] | 52 | 8.57E-06 |
| GO:0035335 | peptidyl-tyrosine dephosphorylation | [None] | 10 | 2.85E-06 |
| GO:0035411 | catenin import into nucleus | [None] | 7 | 3.12E-06 |
| GO:0035412 | regulation of catenin import into nucleus | [None] | 7 | 1.35E-06 |
| GO:0035413 | positive regulation of catenin import into nucleus | [None] | 5 | 3.03E-06 |
| GO:0035556 | intracellular signal transduction | [Module7] | 102 | 7.52E-10 |
| GO:0036211 | protein modification process | [Module1, Module7] | 109 | 9.90E-08 |
| GO:0042060 | wound healing | [None] | 37 | 5.16E-06 |
| GO:0042127 | regulation of cell proliferation | [None] | 58 | 2.79E-05 |
| GO:0042325 | regulation of phosphorylation | [Module11, Module7] | 56 | 2.90E-07 |
| GO:0042327 | positive regulation of phosphorylation | [Module11, Module7] | 44 | 8.30E-08 |
| GO:0042981 | regulation of apoptotic process | [Module12] | 58 | 4.96E-06 |
| GO:0043067 | regulation of programmed cell death | [Module12] | 58 | 8.30E-06 |
| GO:0043408 | regulation of MAPK cascade | [Module7] | 30 | 1.49E-05 |
| GO:0043412 | macromolecule modification | [Module1, Module7] | 112 | 1.04E-07 |
| GO:0043549 | regulation of kinase activity | [Module7] | 39 | 2.34E-06 |
| GO:0044093 | positive regulation of molecular function | [Module11, Module7] | 54 | 3.61E-05 |
| GO:0044249 | cellular biosynthetic process | [Module11] | 174 | 8.85E-06 |
| GO:0044260 | cellular macromolecule metabolic process | [Module13, Module1, Module11] | 225 | 1.98E-07 |
| GO:0044267 | cellular protein metabolic process | [Module13, Module1, Module7] | 127 | 3.75E-06 |
| GO:0045595 | regulation of cell differentiation | [None] | 54 | 3.31E-05 |
| GO:0045859 | regulation of protein kinase activity | [Module7] | 38 | 2.24E-06 |
| GO:0045860 | positive regulation of protein kinase activity | [Module7] | 29 | 2.89E-06 |
| GO:0045937 | positive regulation of phosphate metabolic process | [Module11, Module7] | 45 | 8.89E-07 |
| GO:0048468 | cell development | [Module10, Module8] | 81 | 6.58E-09 |
| GO:0048513 | organ development | [Module10] | 120 | 7.59E-11 |
| GO:0048514 | blood vessel morphogenesis | [Module4] | 31 | 8.72E-07 |
| GO:0048518 | positive regulation of biological process | [Module11] | 148 | 2.58E-07 |
| GO:0048519 | negative regulation of biological process | [Module11] | 147 | 3.09E-12 |
| GO:0048522 | positive regulation of cellular process | [Module11] | 137 | 2.08E-08 |
| GO:0048523 | negative regulation of cellular process | [None] | 137 | 5.53E-12 |
| GO:0048534 | hematopoietic or lymphoid organ development | [None] | 35 | 1.19E-05 |
| GO:0048583 | regulation of response to stimulus | [Module7] | 113 | 2.47E-06 |
| GO:0048585 | negative regulation of response to stimulus | [Module7] | 48 | 1.50E-05 |
| GO:0048666 | neuron development | [Module8] | 44 | 2.62E-05 |
| GO:0048667 | cell morphogenesis involved in neuron differentiation | [Module8] | 34 | 1.42E-05 |
| GO:0048699 | generation of neurons | [Module8] | 55 | 2.11E-05 |
| GO:0048731 | system development | [Module10, Module8] | 162 | 1.97E-14 |
| GO:0048812 | neuron projection morphogenesis | [Module8] | 35 | 8.24E-06 |
| GO:0048858 | cell projection morphogenesis | [Module8] | 39 | 2.13E-05 |
| GO:0048869 | cellular developmental process | [Module10, Module8] | 136 | 6.88E-11 |
| GO:0048870 | cell motility | [None] | 57 | 3.34E-08 |
| GO:0050794 | regulation of cellular process | [Module11, Module2, Module7] | 266 | 1.87E-09 |
| GO:0051049 | regulation of transport | [Module3] | 54 | 1.66E-05 |
| GO:0051171 | regulation of nitrogen compound metabolic process | [Module11] | 132 | 2.86E-05 |
| GO:0051174 | regulation of phosphorus metabolic process | [Module7] | 69 | 5.82E-06 |
| GO:0051246 | regulation of protein metabolic process | [Module1, Module11, Module7] | 75 | 2.70E-07 |
| GO:0051247 | positive regulation of protein metabolic process | [Module11, Module7] | 51 | 1.76E-06 |
| GO:0051252 | regulation of RNA metabolic process | [Module11] | 113 | 1.70E-05 |
| GO:0051338 | regulation of transferase activity | [Module7] | 40 | 1.89E-06 |
| GO:0051347 | positive regulation of transferase activity | [Module7] | 31 | 1.57E-06 |
| GO:0060255 | regulation of macromolecule metabolic process | [Module13, Module11, Module2] | 170 | 3.98E-10 |
| GO:0060341 | regulation of cellular localization | [Module3] | 42 | 1.68E-05 |
| GO:0060429 | epithelium development | [None] | 42 | 9.94E-06 |
| GO:0061008 | hepaticobiliary system development | [None] | 14 | 8.91E-07 |
| GO:0070727 | cellular macromolecule localization | [Module3] | 52 | 1.34E-05 |
| GO:0070887 | cellular response to chemical stimulus | [Module9] | 90 | 1.60E-07 |
| GO:0071310 | cellular response to organic substance | [Module7, Module9] | 77 | 1.20E-07 |
| GO:0071396 | cellular response to lipid | [Module9] | 19 | 4.28E-05 |
| GO:0071702 | organic substance transport | [Module3] | 83 | 2.65E-05 |
| GO:0072358 | cardiovascular system development | [Module4] | 54 | 1.49E-10 |
| GO:0072359 | circulatory system development | [Module4] | 54 | 1.49E-10 |
| GO:0080090 | regulation of primary metabolic process | [Module13, Module11, Module2] | 169 | 5.70E-08 |
| GO:0097194 | execution phase of apoptosis | [None] | 12 | 2.22E-05 |
| GO:0097485 | neuron projection guidance | [Module8] | 24 | 4.02E-05 |
| REACTOME:111045 | Developmental Biology | [Module8] | 28 | 1.15E-06 |
| REACTOME:163936 | Fc epsilon receptor (FCERI) signaling | [Module6, Module9] | 15 | 2.85E-05 |
| GO:1901576 | organic substance biosynthetic process | [Module11] | 179 | 2.69E-06 |
| GO:1901700 | response to oxygen-containing compound | [Module9] | 56 | 4.54E-06 |
| GO:1901701 | cellular response to oxygen-containing compound | [Module9] | 45 | 5.87E-08 |
| GO:1902042 | negative regulation of extrinsic apoptotic signaling pathway via death domain receptors | [None] | 6 | 1.24E-05 |
| GO:1902531 | regulation of intracellular signal transduction | [Module7] | 62 | 1.35E-06 |
| GO:1902533 | positive regulation of intracellular signal transduction | [Module7] | 35 | 3.81E-05 |
| GO:2000112 | regulation of cellular macromolecule biosynthetic process | [Module13, Module11] | 125 | 2.95E-07 |
| GO:2001141 | regulation of RNA biosynthetic process | [Module11] | 111 | 1.36E-05 |
| GO:2001234 | negative regulation of apoptotic signaling pathway | [None] | 15 | 2.67E-05 |
| GO:2001236 | regulation of extrinsic apoptotic signaling pathway | [None] | 15 | 1.56E-05 |
| GO:2001237 | negative regulation of extrinsic apoptotic signaling pathway | [None] | 12 | 3.53E-06 |

supplementary table 4

The function and genes of Modules

| Modules | Function | Module Genes |
| --- | --- | --- |
| Module1 | phosphorylation | ACADVL,ADAM17,AHR,AK2,AKT1S1,AKT3,ALG2,ANAPC7,ANKRD13C,AR,ARAF,ARL6IP5,ASCC2,ATM,B4GALT1,CAV1,CBX2,CCNG1,CDC14B,CDCA7,CDH2,CDK4,CDK6,CEBPA,CHST14,CHSY1,CNKSR3,CNOT6L,COPS2,CPNE3,CREB3L2,CRTC3,CTDSP1,CTDSP2,CTNND1,DACT1,DAPK1,DDIT4,DDX3X,DDX6,DNAJC1,DNM2,DUSP2,E2F1,E2F5,EGFR,EGR1,EIF3B,ELK3,EPHA5,ERG,ETS1,EYA4,EZH2,FBXO11,FBXO33,FLI1,FMR1,FSCN1,FXR1,FZD7,G3BP1,GATA6,GFPT2,GMFB,GNAI2,GNAS,GYS1,H3F3B,HBP1,HECTD2,HECTD3,HIPK3,HIVEP2,HMGB2,HNRNPA3,HTATIP2,IL11,IL6R,INO80C,INPP5D,IQGAP1,IRS1,ITGB1,JARID2,KANK1,KLF6,KRAS,LDLRAP1,LHX2,LITAF,LRIG1,MAGT1,MAN2A1,MAP2K1,MAP3K10,MAP3K13,MAPK14,MEF2A,MEIS1,METAP1,METAP2,MGAT4A,MKX,MORC3,MST4,MTMR6,MYBL1,MYO5A,MYO6,NAA15,NFAT5,NFATC1,NFIA,NKAP,NLK,NOTCH2,NOVA1,NPEPPS,NR3C1,NR4A1,NR6A1,NRAS,NRP1,NSUN2,P4HA1,PAM,PARP16,PARP9,PBX3,PGM1,PGM2,PHF19,PHF6,PIK3C2A,PIP4K2A,PKN2,PLOD3,POU5F1,PPP3CA,PRDM13,PRKAG2,PROX1,PRRX1,PTBP1,PTP4A2,PTPN1,PTPN11,PTPN12,PTPN9,PTPRJ,PTPRZ1,PUM1,PUS3,QKI,QSOX1,RAB27A,RARG,RASSF5,RB1CC1,RELA,RFFL,RHBDF1,RHOG,RLF,RLIM,RNF34,RPS6KA4,RREB1,RYK,SBNO2,SDC4,SEC61B,SERP1,SERPINE1,SERTAD2,SGK1,SHC1,SHPK,SIRT1,SIX4,SLC30A7,SMAD2,SMAD5,SNAI2,SNTA1,SP1,SPHK1,SPI1,SRPK2,STK38,STK4,SUMO1,SWAP70,TAB2,TADA2B,TAOK1,TARBP1,TEAD1,TFEB,TGIF2,TJP2,TLE4,TLN1,TOM1L1,TOMM20,TOR3A,TP53INP1,TRIB3,TRIM32,TSC22D4,TSEN54,TSHZ3,TWSG1,TYK2,UHMK1,USP30,USP38,VAMP3,VKORC1,WEE1,WTAP,WWC1,YES1,ZBTB41,ZBTB6,ZEB2,ZFP36L2,ZIC3,ZMPSTE24,ZNF236,ZNF594,ZNF652 |
| Module2 | cell communication | ABHD5,ACAA2,ACADVL,ADAM17,AHR,AKT1S1,AKT3,ALDH9A1,ALDOA,ANAPC7,ANKRD13C,ANKRD27,AP1G1,AR,ARAF,ARF6,ARFIP1,ARHGEF1,ARL6IP5,ARPC1B,ASCC2,ATM,ATP6V0E1,B4GALT1,C18orf32,CAV1,CBX2,CCNG1,CD164,CD69,CDC14B,CDCA7,CDH2,CDK4,CDK6,CEBPA,CLIC4,CNKSR3,CNOT6L,COPS2,CREB3L2,CRTC3,CTDSP1,CTDSP2,CTNND1,DACT1,DAPK1,DDIT4,DDX3X,DENND5B,DHCR24,DNAJC1,DNM2,DSG2,DUSP2,E2F1,E2F5,EEA1,EGFR,EGR1,EIF3B,ELK3,EPHA5,ERG,ESM1,ETS1,EYA4,EZH2,F11R,FAM129B,FLI1,FLRT3,FMNL2,FMR1,FRMD6,FXR1,FZD7,G3BP1,G3BP2,GATA6,GIGYF1,GMFB,GNAI2,GNAI3,GNAS,GNG10,GPM6B,GSN,HADH,HADHA,HBP1,HIPK3,HIVEP2,HMGB2,HTATIP2,IL11,IL6R,INO80C,INPP5D,IQGAP1,IRS1,ITGA3,ITGB1,ITGB8,ITPRIP,JARID2,KANK1,KATNA1,KCNK2,KIF26A,KLF6,KRAS,LAMC1,LASP1,LDLRAP1,LHX2,LITAF,LRRFIP2,MAN2A1,MAP2K1,MAP3K10,MAP3K13,MAP3K14,MAPK14,MEF2A,MEIS1,METAP1,METAP2,MKX,MORC3,MSN,MST4,MYBL1,MYH10,MYH9,MYO10,MYO5A,MYO6,NAA15,NEDD9,NFAT5,NFATC1,NFIA,NID1,NKAP,NLK,NOTCH2,NOVA1,NR3C1,NR4A1,NR6A1,NRAS,NRP1,OSBPL8,PAM,PARP16,PGRMC2,PHF19,PHF6,PIK3C2A,PKN2,PLCL2,PLEKHM3,PLP2,POU5F1,PPP3CA,PRDM13,PRKAG2,PROX1,PRRX1,PSD3,PTBP1,PTPN1,PTPN11,PTPRJ,PTPRZ1,PUM1,QKI,QSOX1,RAB11FIP5,RAB27A,RAB34,RAB5C,RARG,RASSF5,RB1CC1,RBM24,RDH10,RELA,RFFL,RHBDF1,RHOG,RLF,RLIM,RNF34,RPS6KA4,RRAS,RREB1,RTKN,RYK,SBNO2,SDC4,SEMA5A,SERINC5,SERP1,SERPINE1,SERTAD2,SGK1,SGMS1,SGPL1,SH2B3,SHC1,SHPK,SIRT1,SIX4,SLC29A1,SLC8A3,SLITRK5,SMAD2,SMAD5,SNAI2,SNTA1,SNX18,SOS2,SP1,SPHK1,SPI1,SRGAP1,SRPK2,STAM,STK38,STK4,SUMO1,SYPL1,TAB2,TADA2B,TAOK1,TARBP1,TEAD1,TFEB,TGIF2,TJP2,TLE4,TLN1,TMED1,TMEM109,TMEM64,TMEM9B,TOM1L1,TP53INP1,TRIB3,TRIM2,TRIM32,TSC22D4,TSHZ3,TSKU,TWSG1,TYK2,UHMK1,VAMP3,VIM,WASF2,WEE1,WIPF1,WWC1,YES1,YTHDF2,ZBTB41,ZBTB6,ZEB2,ZFP36L2,ZIC3,ZNF236,ZNF594,ZNF652 |
| Module3 | regulation of localization | ABHD5,ACAA2,ADAM17,ALDOA,ANKRD13C,ANKRD27,ANXA11,AP1G1,AP1M2,AR,ARF6,ARFIP1,ARL6IP5,ARMC1,ATP6V0E1,ATP8A1,B4GALT1,CAV1,CDK6,CLIC4,CLINT1,CNKSR3,COPS2,CPNE3,CREB3L2,CTDSP2,CTNS,CYB5A,DACT1,DAPK1,DHCR24,DNAJC1,DNM2,DSG2,E2F1,EEA1,EGFR,EPHA5,ETS1,FCHO2,FMR1,FRMD6,FZD7,G3BP1,G3BP2,G6PC3,GGA2,GNAI2,GNAI3,GNAS,GPM6B,GSN,HADH,HADHA,HBP1,HTATIP2,IL11,IL6R,IQGAP1,IRS1,ITGA3,ITGB1,KANK1,KATNA1,LASP1,LDLRAP1,LITAF,MAGT1,MAP2K1,MAPK14,MEST,MORC3,MSN,MTMR6,MYH10,MYH9,MYO10,MYO5A,MYO6,NECAP2,NFATC1,NOTCH2,NRP1,OSBP,OSBPL8,PAM,PIK3C2A,PITPNB,PKN2,PLEKHA8,PLEKHF2,PLOD3,PLP2,POU5F1,PPP3CA,PRKAG2,PROX1,PSD3,PTPN1,PTPN11,PTPRJ,PTTG1IP,PUM1,QKI,RAB11FIP5,RAB27A,RAB34,RAB5C,RFFL,RHBDF1,RHOG,RRAS,RYK,RYR3,SCAMP2,SEC61B,SEMA5A,SERP1,SERPINE1,SGK1,SIRT1,SIX4,SLC15A4,SLC16A1,SLC17A5,SLC22A5,SLC25A30,SLC25A39,SLC29A1,SLC30A7,SLC31A2,SLC35F3,SLC7A1,SLC8A3,SLC9A9,SMAD2,SNAI2,SNTA1,SNX16,SNX18,SPDL1,SPHK1,STAM,STX10,SUMO1,TLN1,TMED1,TOM1L1,TOMM20,TP53INP1,TRIB3,TRIM32,UHMK1,UNC119B,VAMP3,VPS37C,WASF2,WIPF1,YES1 |
| Module4 | cardiovascular system development | AHR,ATM,B4GALT1,CAV1,CDH2,CLIC4,COL4A1,EGR1,ELK3,ESM1,ETS1,FZD7,GATA6,GYS1,HTATIP2,ITGA3,ITGB1,ITGB8,MAP2K1,MAPK14,MEF2A,MYH10,MYH9,NAA15,NFATC1,NOTCH2,NR4A1,NRP1,PAM,POU5F1,PROX1,PRRX1,PTPN11,PTPRJ,QKI,RB1CC1,RRAS,SEMA5A,SERPINE1,SGPL1,SHC1,SIRT1,SLITRK5,SMAD2,SNAI2,SPHK1,SPI1,SRPK2,STK4,SUMO1,TAB2,TEAD1,WASF2,ZIC3 |
| Module5 | Glioma | AKT3,ARAF,CDK4,CDK6,CREB3L2,DAPK1,E2F1,EGFR,ETS1,GNAI2,GNAI3,GNAS,KRAS,MAP2K1,NOTCH2,NRAS,SHC1,SOS2,SP1 |
| Module6 | Non-small cell lung cancer | AKT1S1,AKT3,AR,ARAF,ATM,ATP6V0E1,CDK4,CDK6,CEBPA,COL4A1,CREB3L2,DAPK1,DUSP2,E2F1,EGFR,ETS1,FLOT2,FZD7,G6PC3,GNAI2,GNAI3,GNAS,GYS1,IRS1,ITGA3,ITGB1,KANK1,KRAS,LAMC1,MAP2K1,MAP3K13,MAP3K14,MAPK14,NFATC1,NLK,NR4A1,NRAS,OSBPL8,PIK3C2A,PPP3CA,PRKAG2,PTPN1,PTPN11,RASSF5,RELA,RPS6KA4,RRAS,SGK1,SHC1,SIRT1,SMAD2,SOS2,SP1,SPI1,STK4,TAB2,TAOK1,TPM3,TRIB3 |
| Module7 | phosphate-containing compound metabolic process | ABHD5,ACAA2,ACADVL,ADAM17,AHR,AK2,AKT1S1,AKT3,ALDH9A1,ALDOA,ALG2,ANAPC7,ANKRD13C,ANKRD27,AP1G1,AP1M2,AR,ARAF,ARF6,ARFIP1,ARHGEF1,ARL6IP5,ARPC1B,ASCC2,ATM,ATP6V0E1,B4GALT1,C18orf32,CAV1,CBX2,CCNG1,CD164,CD69,CDC14B,CDCA7,CDH2,CDK4,CDK6,CEBPA,CLIC4,CNKSR3,CNOT6L,COL4A1,COPS2,CPNE3,CREB3L2,CRTC3,CTDSP1,CTDSP2,CTNND1,CTNS,DACT1,DAPK1,DDIT4,DDX3X,DENND5B,DHCR24,DNAJC1,DNM2,DSG2,DUSP2,E2F1,E2F5,EEA1,EGFR,EGR1,EIF3B,ELK3,EPHA5,ERG,ESM1,ETS1,EYA4,EZH2,F11R,FAM129B,FAR1,FBXO11,FBXO33,FLI1,FLRT3,FMNL2,FMR1,FRMD6,FXR1,FZD7,G3BP1,G3BP2,G6PC3,GATA6,GFPT2,GIGYF1,GMFB,GNAI2,GNAI3,GNAS,GNG10,GPM6B,GSN,HADH,HADHA,HBP1,HECTD2,HECTD3,HIPK3,HIVEP2,HMGB2,HTATIP2,IL11,IL6R,INO80C,INPP5D,IQGAP1,IRS1,ITGA3,ITGB1,ITGB8,ITPRIP,JARID2,KANK1,KATNA1,KCNK2,KIF26A,KLF6,KRAS,LAMC1,LASP1,LCLAT1,LDLRAP1,LHX2,LITAF,LRRFIP2,MAGT1,MAN2A1,MAP2K1,MAP3K10,MAP3K13,MAP3K14,MAPK14,MEF2A,MEIS1,METAP1,METAP2,MGAT4A,MKX,MORC3,MSN,MST4,MTMR6,MYBL1,MYH10,MYH9,MYO10,MYO5A,MYO6,NAA15,NEDD9,NFAT5,NFATC1,NFIA,NID1,NKAP,NLK,NME4,NOTCH2,NOVA1,NPEPPS,NR3C1,NR4A1,NR6A1,NRAS,NRP1,NSUN2,OSBPL8,P4HA1,PAM,PAPSS2,PARP16,PARP9,PGM2,PGRMC2,PHF19,PHF6,PI4K2B,PIK3C2A,PIP4K2A,PITPNB,PKN2,PLCL2,PLEKHM3,PLOD3,PLP2,PLSCR3,POU5F1,PPP3CA,PRDM13,PRKAG2,PROX1,PRPS1,PRRX1,PSD3,PTBP1,PTP4A2,PTPN1,PTPN11,PTPN12,PTPN9,PTPRJ,PTPRZ1,PUM1,PUS3,QKI,QSOX1,RAB11FIP5,RAB27A,RAB34,RAB5C,RARG,RASSF5,RB1CC1,RBM24,RDH10,RELA,RFFL,RHBDF1,RHOG,RLF,RLIM,RNF34,RPS6KA4,RRAS,RREB1,RTKN,RYK,RYR3,SBNO2,SDC4,SEC61B,SEMA5A,SERINC5,SERP1,SERPINE1,SERTAD2,SGK1,SGMS1,SGPL1,SH2B3,SHC1,SHPK,SIRT1,SIX4,SLC16A1,SLC29A1,SLC30A7,SLC8A3,SLITRK5,SMAD2,SMAD5,SNAI2,SNTA1,SNX18,SOS2,SP1,SPHK1,SPI1,SRGAP1,SRPK2,STAM,STK38,STK4,SUMO1,SYPL1,TAB2,TADA2B,TAOK1,TARBP1,TEAD1,TFEB,TGIF2,TJP2,TLE4,TLN1,TMED1,TMEM109,TMEM64,TMEM9B,TOM1L1,TOMM20,TOR3A,TP53INP1,TRIB3,TRIM2,TRIM32,TSC22D4,TSHZ3,TSKU,TWSG1,TYK2,UHMK1,USP30,USP38,VAMP3,VIM,VKORC1,WASF2,WEE1,WIPF1,WWC1,YES1,ZBTB41,ZBTB6,ZEB2,ZFP36L2,ZIC3,ZMPSTE24,ZNF236,ZNF594,ZNF652 |
| Module8 | multicellular organismal development | ABHD5,ACADVL,ADAM17,AHR,AK2,AKT3,ALDH9A1,ALDOA,AR,ARF6,ARHGEF1,ATM,B4GALT1,BTBD3,CAV1,CBX2,CCNG1,CD164,CDH2,CDK4,CDK6,CEBPA,CLIC4,COL4A1,COPS2,CREB3L2,CTDSP1,CTNND1,CTNS,DACT1,DDIT4,DHCR24,DNM2,DUSP2,E2F1,E2F5,EGFR,EGR1,ELK3,EPHA5,ERG,ESM1,ETS1,EVI5,EYA4,EZH2,F11R,FLI1,FLOT2,FLRT3,FMNL2,FMR1,FPGS,FRMD6,FXR1,FZD7,GATA6,GMFB,GNAI2,GNAS,GPM6B,GSN,GYS1,H3F3B,HMGB2,HTATIP2,IL11,IL6R,INPP5D,IQGAP1,IRS1,ITGA3,ITGB1,ITGB8,JARID2,KANK1,KATNA1,KIF26A,KLF6,KRAS,LAMC1,LCLAT1,LHX2,LRIG1,MAN2A1,MAP2K1,MAPK14,MEF2A,MEIS1,METAP2,MKX,MORC3,MSN,MST4,MYH10,MYH9,MYO10,MYO5A,MYO6,NAA15,NFATC1,NID1,NKAP,NOTCH2,NR3C1,NR4A1,NRAS,NRP1,OSBPL8,PAM,PAPSS2,PBX3,PCDH9,PCDHA2,PHF19,PIP4K2A,PITPNB,PKN2,PLOD3,POU5F1,PPP3CA,PRDM13,PROX1,PRPS1,PRRX1,PSD3,PTBP1,PTPN11,PTPRJ,PTPRZ1,PTTG1IP,QKI,RAB27A,RARG,RB1CC1,RBM24,RDH10,RELA,RHOG,RPS6KA4,RRAS,RREB1,RYK,SDC4,SEMA5A,SERINC5,SERP1,SERPINE1,SGPL1,SH2B3,SHC1,SIRT1,SIX4,SLC29A1,SLC8A3,SLITRK4,SLITRK5,SMAD2,SMAD5,SNAI2,SOS2,SP1,SPHK1,SPI1,SRGAP1,SRPK2,STK4,SUMO1,SWAP70,TAB2,TAGLN2,TEAD1,TFEB,TLN1,TMEM64,TRIB3,TRIM32,TSHZ3,TSKU,TTC7A,TWSG1,UHMK1,UNC119B,VAMP3,VANGL1,VIM,WASF2,WEE1,ZEB2,ZFP36L2,ZIC3 |
| Module9 | cellular response to oxygen-containing compound | ACADVL,ADAM17,AHR,AK2,AKT1S1,AKT3,AR,ARHGEF1,ARL6IP5,ATP6V0E1,B4GALT1,CAV1,CCNG1,CD69,CDH2,CDK4,CEBPA,CLIC4,CNKSR3,COL4A1,CREB3L2,CTDSP2,CTNND1,DACT1,DAPK1,DDIT4,DDX3X,DHCR24,DSG2,E2F1,E2F5,EGFR,EGR1,EPHA5,ESM1,ETS1,F11R,FZD7,G3BP1,GATA6,GIGYF1,GNAI2,GNAI3,GNAS,GNG10,GSN,H3F3B,HADH,HADHA,HBP1,HMGB2,IL6R,INPP5D,IQGAP1,IRS1,ITGA3,ITGB1,ITGB8,ITPRIP,KANK1,KCNK2,KLF6,KRAS,LITAF,LRIG1,LRRFIP2,MAP2K1,MAP3K10,MAPK14,MEF2A,MEST,METAP1,METAP2,MYH9,MYO10,MYO5A,NEDD9,NFATC1,NFIA,NKAP,NLK,NOTCH2,NPEPPS,NR4A1,NRAS,NRP1,OSBPL8,PAM,PAPSS2,PARP16,PARP9,PGRMC2,PIK3C2A,PKN2,PLOD3,PLP2,PLSCR3,POU5F1,PPP3CA,PRKAG2,PRRX1,PTPN1,PTPN11,PTPRJ,RAB11FIP5,RARG,RB1CC1,RELA,RFFL,RHBDF1,RHOG,RNF34,RPS6KA4,RYK,RYR3,SEMA5A,SERP1,SERPINE1,SGMS1,SGPL1,SHC1,SHPK,SIRT1,SLC16A1,SLC29A1,SLC8A3,SMAD2,SMAD5,SNAI2,SOS2,SP1,SPHK1,STAM,STK4,SUMO1,TAB2,TGIF2,TJP2,TLE4,TLN1,TP53INP1,TRIB3,TRIM32,TSKU,TWSG1,TYK2,WASF2,WIPF1,YES1,ZEB2,ZNF236 |
| Module10 | system development | ABHD5,ACADVL,ADAM17,AHR,AK2,ALDH9A1,ALDOA,AR,ARF6,ARHGEF1,ATM,B4GALT1,BTBD3,CAV1,CBX2,CCNG1,CD164,CDH2,CDK4,CDK6,CEBPA,CLIC4,COL4A1,COPS2,CREB3L2,CTDSP1,CTNND1,CTNS,DACT1,DDIT4,DHCR24,DUSP2,E2F1,E2F5,EGFR,EGR1,ELK3,EPHA5,ERG,ESM1,ETS1,EVI5,EYA4,EZH2,F11R,FLI1,FLOT2,FLRT3,FMNL2,FMR1,FPGS,FRMD6,FXR1,FZD7,GATA6,GMFB,GNAI2,GNAS,GPM6B,GSN,GYS1,H3F3B,HMGB2,HTATIP2,IL11,IL6R,INPP5D,IQGAP1,IRS1,ITGA3,ITGB1,ITGB8,JARID2,KANK1,KATNA1,KIF26A,KLF6,KRAS,LAMC1,LCLAT1,LHX2,LRIG1,MAN2A1,MAP2K1,MAPK14,MEF2A,MEIS1,METAP2,MKX,MORC3,MSN,MST4,MYH10,MYH9,MYO10,MYO5A,MYO6,NAA15,NFATC1,NID1,NKAP,NOTCH2,NR3C1,NR4A1,NRAS,NRP1,OSBPL8,PAM,PAPSS2,PBX3,PCDH9,PCDHA2,PHF19,PIP4K2A,PITPNB,PKN2,PLOD3,POU5F1,PPP3CA,PRDM13,PROX1,PRPS1,PRRX1,PSD3,PTBP1,PTPN11,PTPRJ,PTPRZ1,PTTG1IP,QKI,RAB27A,RARG,RB1CC1,RBM24,RDH10,RELA,RHOG,RPS6KA4,RRAS,RREB1,RYK,SDC4,SEMA5A,SERINC5,SERP1,SERPINE1,SGPL1,SH2B3,SHC1,SIRT1,SIX4,SLC29A1,SLC8A3,SLITRK4,SLITRK5,SMAD2,SMAD5,SNAI2,SOS2,SP1,SPHK1,SPI1,SRGAP1,SRPK2,STK4,SUMO1,SWAP70,TAB2,TAGLN2,TEAD1,TFEB,TLN1,TMEM64,TRIB3,TRIM32,TSHZ3,TSKU,TTC7A,TWSG1,UHMK1,UNC119B,VAMP3,VANGL1,VIM,WASF2,WEE1,ZEB2,ZFP36L2,ZIC3 |
| Module11 | negative regulation of biological process | ABHD5,ACAA2,ACADVL,ADAM17,AHR,AK2,AKT1S1,AKT3,ALDH9A1,ALDOA,ALG2,ANAPC7,ANKRD13C,ANKRD27,AP1G1,AR,ARAF,ARF6,ARFIP1,ARHGEF1,ARL6IP5,ARPC1B,ASCC2,ATM,ATP6V0E1,B4GALT1,C18orf32,CAV1,CBX2,CCNG1,CD164,CD69,CDC14B,CDCA7,CDH2,CDK4,CDK6,CEBPA,CHST14,CHSY1,CLIC4,CNKSR3,CNOT6L,COPS2,CPNE3,CREB3L2,CRTC3,CTDSP1,CTDSP2,CTNND1,DACT1,DAPK1,DDIT4,DDX3X,DDX6,DENND5B,DHCR24,DNAJC1,DNM2,DSG2,DUSP2,E2F1,E2F5,EGFR,EGR1,EIF3B,ELK3,EPHA5,ERG,ESM1,ETS1,EYA4,EZH2,F11R,FAM129B,FAR1,FBXO11,FBXO33,FLI1,FLRT3,FMNL2,FMR1,FRMD6,FSCN1,FXR1,FZD7,G3BP1,G3BP2,G6PC3,GATA6,GFPT2,GIGYF1,GMFB,GNAI2,GNAI3,GNAS,GNG10,GPM6B,GSN,GYS1,H3F3B,HADH,HADHA,HBP1,HECTD2,HECTD3,HIPK3,HIVEP2,HMGB2,HNRNPA3,HTATIP2,IL11,IL6R,INO80C,INPP5D,IQGAP1,IRS1,ITGA3,ITGB1,ITGB8,ITPRIP,JARID2,KANK1,KATNA1,KCNK2,KIF26A,KLF6,KRAS,LAMC1,LASP1,LCLAT1,LDLRAP1,LHX2,LITAF,LRIG1,LRRFIP2,MAGT1,MAN2A1,MAP2K1,MAP3K10,MAP3K13,MAP3K14,MAPK14,MEF2A,MEIS1,METAP1,METAP2,MGAT4A,MKX,MORC3,MSN,MST4,MTMR6,MYBL1,MYH10,MYH9,MYO10,MYO5A,MYO6,NAA15,NEDD9,NFAT5,NFATC1,NFIA,NID1,NKAP,NLK,NME4,NOTCH2,NOVA1,NPEPPS,NR3C1,NR4A1,NR6A1,NRAS,NRP1,NSUN2,OSBPL8,P4HA1,PAM,PAPSS2,PARP16,PARP9,PBX3,PGM1,PGM2,PGRMC2,PHF19,PHF6,PI4K2B,PIK3C2A,PIP4K2A,PITPNB,PKN2,PLCL2,PLEKHM3,PLOD3,PLP2,POU5F1,PPP3CA,PRDM13,PRKAG2,PROX1,PRPS1,PRRX1,PSD3,PTBP1,PTP4A2,PTPN1,PTPN11,PTPN12,PTPN9,PTPRJ,PTPRZ1,PUM1,PUS3,QKI,QSOX1,RAB11FIP5,RAB27A,RAB34,RAB5C,RARG,RASSF5,RB1CC1,RBM24,RDH10,RELA,RFFL,RHBDF1,RHOG,RLF,RLIM,RNF34,RNPEPL1,RPS6KA4,RRAS,RREB1,RTKN,RYK,SBNO2,SCD,SDC4,SEC61B,SEMA5A,SERINC5,SERP1,SERPINE1,SERTAD2,SGK1,SGMS1,SGPL1,SH2B3,SHC1,SHPK,SIRT1,SIX4,SLC22A5,SLC25A39,SLC30A7,SMAD2,SMAD5,SNAI2,SNTA1,SNX18,SOS2,SP1,SPHK1,SPI1,SRGAP1,SRPK2,STAM,STK38,STK4,SUMO1,SWAP70,TAB2,TADA2B,TAOK1,TARBP1,TEAD1,TFEB,TGIF2,TJP2,TLE4,TLN1,TMED1,TMEM109,TMEM64,TMEM9B,TOM1L1,TOMM20,TOR3A,TP53INP1,TRIB3,TRIM2,TRIM32,TSC22D4,TSEN54,TSHZ3,TSKU,TWSG1,TYK2,UHMK1,USP30,USP38,VAMP3,VIM,VKORC1,VPS37C,WASF2,WEE1,WIPF1,WTAP,WWC1,YES1,YTHDF2,ZBTB41,ZBTB6,ZEB2,ZFP36L2,ZIC3,ZMPSTE24,ZNF236,ZNF594,ZNF652 |
| Module12 | programmed cell death | ACAA2,ADAM17,AHR,AKT1S1,ANKRD13C,AR,ARAF,ARF6,ARL6IP5,ATM,B4GALT1,C3orf38,CAV1,CCNG1,CDCA7,CDK4,CNKSR3,DAPK1,DDIT4,DDX3X,DHCR24,DNM2,DSG2,DUSP2,E2F1,EGFR,EGR1,ETS1,FAM129B,FXR1,GATA6,GSN,HIPK3,HMGB2,HTATIP2,IL6R,INPP5D,ITGB1,ITPRIP,KRAS,LITAF,MAP3K10,MAPK14,MEF2A,MST4,NAA15,NOTCH2,NR3C1,NR4A1,NRAS,NRP1,PARP16,PKM,PKN2,PLSCR3,RARG,RASSF5,RB1CC1,RELA,RFFL,RNF34,RTKN,SEMA5A,SERPINE1,SGK1,SGMS1,SGPL1,SIRT1,SIX4,SNAI2,SOS2,SPHK1,SPI1,SRPK2,STK4,TAOK1,TJP2,TMEM109,TP53INP1,TRIB3,TRIM2,TRIM32,VIM |
| Module13 | regulation of macromolecule metabolic process | ABHD5,ACADVL,ADAM17,AHR,AKT1S1,AKT3,ALG2,ANAPC7,ANKRD13C,ANKRD27,AR,ARAF,ARHGEF1,ARL6IP5,ASCC2,ATM,B4GALT1,CAV1,CBX2,CCNG1,CDC14B,CDCA7,CDH2,CDK4,CDK6,CEBPA,CHST14,CHSY1,CNKSR3,CNOT6L,COPS2,CPNE3,CREB3L2,CRTC3,CTDSP1,CTDSP2,CTNND1,DACT1,DAPK1,DDIT4,DDX3X,DDX6,DENND5B,DHCR24,DNAJC1,DNM2,DUSP2,E2F1,E2F5,EGFR,EGR1,EIF3B,ELK3,EPHA5,ERG,ETS1,EYA4,EZH2,FBXO11,FBXO33,FLI1,FMR1,FSCN1,FXR1,FZD7,G3BP1,GATA6,GFPT2,GMFB,GNAI2,GNAI3,GNAS,GYS1,H3F3B,HBP1,HECTD2,HECTD3,HIPK3,HIVEP2,HMGB2,HNRNPA3,HTATIP2,IL11,IL6R,INO80C,INPP5D,IQGAP1,IRS1,ITGA3,ITGB1,JARID2,KANK1,KLF6,KRAS,LDLRAP1,LHX2,LITAF,LRIG1,MAGT1,MAN2A1,MAP2K1,MAP3K10,MAP3K13,MAPK14,MEF2A,MEIS1,METAP1,METAP2,MGAT4A,MKX,MORC3,MSN,MST4,MTMR6,MYBL1,MYH9,MYO5A,MYO6,NAA15,NFAT5,NFATC1,NFIA,NKAP,NLK,NOTCH2,NOVA1,NPEPPS,NR3C1,NR4A1,NR6A1,NRAS,NRP1,NSUN2,P4HA1,PAM,PARP16,PARP9,PBX3,PGM1,PGM2,PHF19,PHF6,PKN2,PLOD3,POU5F1,PPP3CA,PRDM13,PRKAG2,PROX1,PRRX1,PSD3,PTBP1,PTP4A2,PTPN1,PTPN11,PTPN12,PTPN9,PTPRJ,PTPRZ1,PUM1,PUS3,QKI,QSOX1,RAB27A,RARG,RASSF5,RB1CC1,RBM24,RELA,RFFL,RHBDF1,RHOG,RLF,RLIM,RNF34,RNPEPL1,RPS6KA4,RREB1,RTKN,RYK,SBNO2,SDC4,SEC61B,SEMA5A,SERINC5,SERP1,SERPINE1,SERTAD2,SGK1,SGMS1,SHC1,SIRT1,SIX4,SLC30A7,SMAD2,SMAD5,SNAI2,SNTA1,SNX18,SP1,SPHK1,SPI1,SRPK2,STK38,STK4,SUMO1,SWAP70,TAB2,TADA2B,TAOK1,TARBP1,TEAD1,TFEB,TGIF2,TLE4,TLN1,TMEM64,TOM1L1,TOMM20,TOR3A,TP53INP1,TRIB3,TRIM32,TSC22D4,TSEN54,TSHZ3,TSKU,TWSG1,TYK2,UHMK1,USP30,USP38,VAMP3,VKORC1,VPS37C,WEE1,WTAP,WWC1,YES1,YTHDF2,ZBTB41,ZBTB6,ZEB2,ZFP36L2,ZIC3,ZMPSTE24,ZNF236,ZNF594,ZNF652 |

supplementary table 5

The primer of mRNAs and miRNAs

| mRNA |  | |
| --- | --- | --- |
| Gene symbol | Primer sequence(5’ → 3’) | |
| NRAS_F_1 | CCATTCCAGAGCTTGTGAGC | |
| NRAS_R_1 | ACAAGAAGCAGAACGCACCA | |
| KRAS_F_1 | GTTTGAAGTGCCTGTTTGGGA | |
| KRAS_R_1 | CAGTTAGCTCTGTGGGGGTG | |
| VIM_F_1 | CTGCCAACCGGAACAATGAC | |
| VIM_R_1 | CATTTCACGCATCTGGCGTT | |
| ESM1_F_1 | GGGAGCTAGGCAAAGCTGAA | |
| ESM1_R_1 | GCTACCTACCAAGGAAGGGC | |
| GAPDH_F_1 | CCAGCAAGAGCACAAGAGGAA | |
| GAPDH_R_1 | CAAGGGGTCTACATGGCAACT | |
| miRNA |  | |
| hsa-miR-181d | MIMAT0002821 Rtprimer | GTTGGCTCTGGTGCAGGGTCCGAGGTATTCGCACCAGAGCCAACACCCAC |
|  | MIMAT0002821 Forward primer | CGGCGGAACATTCATTGTTGTCG |
| hsa-miR-181b-3p | MIMAT0022692 RTprimer | GTTGGCTCTGGTGCAGGGTCCGAGGTATTCGCACCAGAGCCAACTTGCAT |
|  | MIMAT0022692 Forward primer | CGGCGGCTCACTGAACAATGA |
| hsa-miR-181b-5p | MIMAT0000257 Rtprimer | GTTGGCTCTGGTGCAGGGTCCGAGGTATTCGCACCAGAGCCAACACCCAC |
|  | MIMAT0000257 Forward primer | CGGCGGAACATTCATTGCTGTCG |
| hsa-miR-605-3p | MIMAT0026621 RTprimer | GTTGGCTCTGGTGCAGGGTCCGAGGTATTCGCACCAGAGCCAACTCTAAA |
|  | MIMAT0026621 Forward primer | CGGCGGAGAAGGCACTATGAGA |
